# A Hybrid Grey Wolf Optimizer for Process Planning Optimization with Precedence Constraints

**DOI:** 10.3390/ma14237360

**Published:** 2021-11-30

**Authors:** Mijodrag Milosevic, Robert Cep, Lenka Cepova, Dejan Lukic, Aco Antic, Mica Djurdjev

**Affiliations:** 1 Department of Production Engineering, Faculty of Technical Sciences, University of Novi Sad, 21000 Novi Sad, Serbia; mido@uns.ac.rs (M.M.); lukicd@uns.ac.rs (D.L.); antica@uns.ac.rs (A.A.); 2 Department of Machining, Assembly and Engineering Metrology, Faculty of Mechanical Engineering, Technical University of Ostrava, 70800 Ostrava, Czech Republic; robert.cep@vsb.cz (R.C.); lenka.cepova@vsb.cz (L.C.); 3 Department of Mechanical Engineering, Technical Faculty “Mihajlo Pupin”, University of Novi Sad, 23000 Zrenjanin, Serbia

**Keywords:** process planning optimization, grey wolf optimizer, precedence constraints, constraint handling, selection, crossover, mutation

## Abstract

Process planning optimization is a well-known NP-hard combinatorial problem extensively studied in the scientific community. Its main components include operation sequencing, selection of manufacturing resources and determination of appropriate setup plans. These problems require metaheuristic-based approaches in order to be effectively and efficiently solved. Therefore, to optimize the complex process planning problem, a novel hybrid grey wolf optimizer (HGWO) is proposed. The traditional grey wolf optimizer (GWO) is improved by employing genetic strategies such as selection, crossover and mutation which enhance global search abilities and convergence of the traditional GWO. Precedence relationships among machining operations are taken into account and precedence constraints are modeled using operation precedence graphs and adjacency matrices. Constraint handling heuristic procedure is adopted to move infeasible solutions to a feasible domain. Minimization of the total weighted machining cost of a process plan is adopted as the objective and three experimental studies that consider three different prismatic parts are conducted. Comparative analysis of the obtained cost values, as well as the convergence analysis, are performed and the HGWO approach demonstrated effectiveness and flexibility in finding optimal and near-optimal process plans. On the other side, comparative analysis of computational times and execution times of certain MATLAB functions showed that the HGWO have good time efficiency but limited since it requires more time compared to considered hybrid and traditional algorithms. Potential directions to improving efficiency and performances of the proposed approach are given in conclusions.

## 1. Introduction

Process planning optimization (PPO) problems consist of two subproblems: operations selection and operations sequencing [1]. Operations selection is a task of selecting necessary machining operations for each machining feature recognized on a part or a product. In order to perform these operations, adequate machining resources are required. In that sense, the PPO problem includes selection of machine candidate, cutting tool candidate and tool approach direction (TAD) candidate for each machining operation. On the other hand, operations sequencing represents a task of finding the order of selected machining operations with respect to the predetermined precedence constraints based on relationships between machining features. Tackling with such a problem requires dealing with a number of alternatives which makes the PPO a combinatorial optimization problem. A number of alternative process plans grows in accordance with problem dimensions. In other words, as a number of machining features increases, so does a number of machining operations. This leads to exponential time growth required to find optimal or near-optimal process plans, which means that the PPO problem belongs to the class of NP-hard (non-deterministic polynomial) optimization problems.

As it is impossible to check every possible solution to an NP-hard problem, conventional non-heuristic methods have proved to be ineffective. The PPO problem requires the implementation of metaheuristic algorithms which have shown promising performance for solving difficult problems. This is especially due to their ability to achieve a trade-off between local and global search [2]. This paper proposes a novel hybrid grey wolf optimizer (HGWO) to deal with the PPO problem with precedence constraints. The manuscript is organized as follows. Section 2 provides recent studies related to the PPO. Section 3 describes the problem, focusing on manufacturing interactions and precedence constraints, representation of process plans and mathematical modelling of the PPO. Section 4 emphasizes the proposed HGWO approach starting from traditional GWO, then genetic strategies that are included, and constraint handling algorithm as part of the structure of the HGWO. Section 5 represents the experimental research that consider three experimental studies, or three prismatic parts. With detailed description of these cases, comparative analysis of cost values and convergence curves are performed to test the performances of the HGWO. Later, discussion in Section 6, emphasize the effectiveness of the proposed method and the computational efficiency. Conclusions with directions for future research are given in Section 7.

## 2. Related Research Studies

In the past few decades, many search algorithms have been employed to deal with the PPO problem. In the initial stages of research on the PPO, genetic algorithms have been widely reported among other metaheuristics. One of the earliest implementations of genetic algorithms (GAs) can be found in Zhang et al. [3]. Besides the GA, many other algorithms such as ACO, PSO, TS, SA are applied to this day.

GA it is the most popular evolutionary algorithm based on a Darwinian theory of natural selection and genetics. Traditional GAs, as well as many other algorithms, require additional modifications to improve their convergence as well as global and local search abilities. Study performed in Salehi and Bahreininejad [4] adopts hybrid GA and intelligent search for optimization of process planning in preliminary and detailed planning stages. The initial and feasible solutions were checked by order and clustering constraints. Li et al. [5] proposed a hybridization model of GA and SA where the initial process plans were generated using the GA approach while the optimal or near-optimal process plans were obtained using the SA algorithm. Kafashi [6] developed a GA approach in order to tackle integrated setup planning and operation sequencing problem in flexible manufacturing environment. With the emphasis on technological constraints, tolerance relation analysis and feature-based representation, satisfactory setup plans and operation sequences were generated. Cai et al. [7] used the GA approach based on optimization toolbox to address adaptive setup planning problem towards the process planning and scheduling integration. They considered machine availability based on tool accessibility analysis as a constraint, and selected certain scheduling criteria such as cost, makespan or machine utilization. Huang et al. [8] proposed a hybrid graph and GA approach. Combining graph theory as well as matrix theory, precisely operation precedence graphs and adjacency matrices, precedence constraints were formulated and a GA approach based on modified crossover and mutation strategies were utilized to solve the PPO problem. Two heuristic approaches are incorporated within this hybrid approach in order to adjust infeasible process plans to a feasible domain. Li et al. [9] employed a novel two phase genetic algorithm to optimize process parameters and machining sequence for two-tool parallel drilling operations with multiple blind holes distributed in a pair of parallel faces and in multiple pairs of parallel faces. Hybridization of GA and local search for the PPO on turning machine tool was proposed by Su et al. [10]. They formulated the PPO problem as a mixed 0–1 integer programming model and used a novel encoding strategy to deal with complicated precedence constraints. In the study presented by Su et al. [11], precedence constrained operation sequencing problem was formulated and edge selection based strategy was adopted to assure feasibility of solutions and improve GA’s converging efficiency. Dou et al. [12] proposed the improved GA by considering fragment crossover and mutation operators with adaptive operation probabilities as well as a new elitist-based crossover strategy. Luo et al. [13] dealt with a large-sized process sequencing problem with complex association constraints. The problem with huge and complex solution space was decomposed to smaller multi-neighborhood spaces and hybrid GA and variable neighborhood search (VNS) approach were used to obtain the best solutions through all neighborhood spaces.

Furthermore, the ant colony optimization algorithm also found its application to the PPO. Liu et al. [14] used ant colony optimization for process planning optimization. They based their research on mapping the PPO problem to a weighted graph and converting to a constraint-based travelling salesman problem. Constraint and state matrices were used to ensure the feasibility of process plans. Wang et al. [15] dealt with the PPO problem by using a weighted directed graph for process plan representation. Wang et al. [16] proposed a two-stage ACO algorithm to solve the PPO problem for prismatic parts. The PPO was formulated using a directed graph and then the proposed ACO approach was used for optimizing process plans for two prismatic parts from the literature. First stage included operation selection while the second was focused on operation sequencing. Hu et al. [17] considered precedence constraint and clustering constraint relationships to ensure feasible permutations and enhanced search abilities. They used the updating method and the local search mechanism to modify the ACO algorithm.

Particle swarm optimization (PSO) is also one of the most popular metaheuristics based on swarm intelligence. This method is inspired by social behavior of living organisms that live in groups (swarms), such as birds or fish [18]. One of the recent studies concerning the PSO in process planning can be found in [19]. Here, authors proposed a modified PSO algorithm improved by adopting efficient encoding, updating and random search methods in order to tackle seven different case studies that consider prismatic parts. Petrović et al. [20] developed a new chaotic PSO algorithm for flexible process planning. By emphasizing different types of flexibilities, authors used AND/OR networks to represent process plans and then tested performances of the cPSO on several cases including cylindrical as well as prismatic parts. Miljković and Petrović [21] developed a modified multi-objective PSO algorithm for flexible process planning. By emphasizing different types of flexibilities, authors used AND/OR networks to represent feasible process plans, and tested performances of the cPSO on several cases including cylindrical as well as prismatic parts. Dou et al. [22] proposed a novel feasible sequence-oriented discrete PSO algorithm to solve the operation sequencing problems in CAPP. It incorporates novel crossover and mutation operators with adaptive probabilities to evolve particles and improve exploration ability.

Lian et al. [23] proposed the imperialist competitive algorithm to address the PPO problem. This socio-politically motivated population-based metaheuristic was inspired by imperialist competition. The approach utilizes steps of assimilation, competition, revolution and elimination to obtain optimal process plans according to predefined networks and types of flexibilities.

Wen et al. [24] proposed a new method based on honey bees mating optimization (HBMO) algorithm to optimize the PPO. The solution encoding, crossover operator and local search strategies were developed, and three experiments were carried out to demonstrate improvement of the HBMO approach.

A new heuristic method, called cross entropy approach was proposed to optimize flexible process planning by Lv and Qiao [25]. Its authors adopted AND/OR networks to represent flexible process plans and established mathematical model for the minimization of total processing time and total cost of flexible process plans. The new sample representation and probability distribution parameter were introduced and case studies were carried and discussed to indicate the performance and adaptability of the CE approach.

Wang et al. [26] adopted a hybrid bat algorithm for the PPO focusing on both crucial tasks, operations selection and operations sequencing. Encoding, decoding and initialization strategies were proposed and two local search strategies were incorporated into the standard bat algorithm (BA) to improve its local convergence. A classical simulation experiment was conducted to verify the validity of the hybrid BA.

Musharavati and Hamouda [27] investigated the possibility of implementing four different configurations of simulated annealing algorithm to solve the process planning problem in reconfigurable manufacturing systems. They used knowledge exploitation and quasi-parallelism as concepts to enhance the SA algorithms. Performances of the variant algorithms are compared and ANOVA methodology is used in computational analysis to test the means and indicate improvements towards better optimal solutions.

Mohammadi et al. [28] dealt with the multi-objective optimization of the integrated process planning and scheduling problem. They designed a slot-based mixed integer linear programming model that accounts for sequence-dependent preparation times. Minimization of manufacturing cost in process planning, and minimization of preparation times and total tardiness were considered as optimization objectives. To solve the problem, a hybrid simulated annealing approach was introduced. The SA was modified with tabu search algorithm as well as local search strategies in order to improve solution’s quality and avoid premature convergence.

Xu et al. [29] introduced a novel NC process reuse-oriented flexible process planning optimization approach for prismatic parts with the objective of minimizing total manufacturing cost. A hybrid ant colony algorithm (ACA) and simulated annealing (SA) approach based on operation precedence graph (OPG) was presented to find the global optimal NC process scheme for the part.

Lian et al. [30] proposed a multi-dimensional tabu search algorithm to optimize four dimensions of a process plan, such as operation sequence, machine sequence, tool sequence, and tool approach direction sequence. Classical neighborhood strategies such as insertion and swap are used to improve the performance of MDTS.

Falih and Shammari [31] proposed a novel hybrid constrained permutation algorithm and genetic algorithm approach for process planning problem. A set of feasible operation sequences is generated using a CPA algorithm and the GA with mixed crossover operator is further employed to search for optimal or near optimal process plans.

Gao et al. [32] suggested the intelligent water drop algorithm for solving the process planning problem. Firstly, operation units were defined according to the processing characteristics, and later, the IWD algorithm was combined with the process planning problem.

Kizys et al. [33] developed an iterative local search metaheuristic and quadratic programming approach to deal with variants of the mean-variance portfolio optimization problem subject to cardinality and quantity constraints.

Sawik and Sawik [34] used stochastic programming approach to optimize cybersecurity investment in supply chains. A mixed binary optimization problem is transformed to an unconstrained binary program in order to maximize total cybersecurity value of control portfolio. It is also shown that the portfolio of security controls with maximum total cybersecurity value reduces the losses from security breaches and mitigates the impact of cyber risk.

In one of the recent studies, Milosević et al. [35] presented the intelligent process planning for the concepts of smart factory and smart manufacturing. Nature-inspired metaheuristic algorithms as smart services of artificial intelligence for intelligent process planning optimization within smart manufacturing were proposed. Three modern algorithms such as grey wolf optimizer (GWO), whale optimization algorithm (WOA) and crow search algorithm (CSA) were employed on three classical case studies from the literature.

Djurdjev et al. [36] proposed a novel genetic crow search approach (GCSA) for operation sequencing in process planning. The traditional CSA algorithm has been improved by adopting genetic strategies such as tournament selection, three-string crossover, shift mutation and resource mutation. Adaptive probabilities were introduced to improve local and global capabilities of the GCSA. Besides, the nearest mechanism strategy was added to ensure feasibility of machine, tools and TADs vectors. Lastly, repairment heuristic strategy was used to handle precedence constraints among features and machining operations.

Aforementioned studies have shown that the improvements made by authors largely increased efficiency of metaheuristic algorithms. However, as stated in [20], main drawbacks reflect in time-consuming optimization and slow convergence of TS, SA and GA approaches particularly. The key and also the most challenging task in the implementation of metaheuristic algorithms is to find a proper balance between exploration and exploitation phases [37], and thereby, maintain its simplicity and flexibility. Many different concepts have been developed and tested so far, making them available for application in different scientific fields. In this research, we focus on implementing novel metaheuristic approach to solve the PPO problem.

## 3. Process Planning Optimization Problem

Computer-aided process planning represents the link between computer-aided design and computer-aided manufacturing within the computer integrated environment. The aim of process planning is to manufacture a part or a product starting from its initial design stages to its final stage (a finished part or product). The input to process planning includes valuable geometric information obtained from a CAD file. Accordingly, process planners have a goal to map product information to a technological domain. Parts/products are generally described by design features which are characterized by geometric forms or shapes with technological attributes such as tolerances and surface finishes. Design features are transformed to manufacturing features which represent manufacturing meaning of the geometry of a part, a product or an assembly associated with certain manufacturing activities [38]. An important subset of manufacturing features consists of machining features such as holes, steps, grooves, pockets, planes, etc. Recognized machining features are most often considered as an input information for the PPO problem.

Apart from machining features that require certain operations in order to be machined on a part or a product, process planning considers other activities such as finding the sequence of machining operations, determining machining resources—machine tools, cutting tools, fixtures, determining cutting conditions and calculating machining time and cost. In the PPO, the quality of a single process plan is determined by selecting machining resources such as machine tools, cutting tools, and tool approach directions (TADs) for each machining operation, as well as generating optimal sequence of machining operations for an observed part or a product. During the decision-making process of selecting alternatives of machining resources and TADs, in order to find feasible and optimal order of machining operations, the sequence have to satisfy precedence constraints that are based on relationships between machining features and machining operations.

### 3.1. Representation of Process Plans

When dealing with the PPO problem, the first step is to select an appropriate representation of a process plan. In recent studies [20,21,23,25] five types of flexibilities were considered, and AND/OR networks are used to represent the PPO problem. The main advantage of these networks is in the fact that a single AND/OR network visualizes the detailed representation of all flexibilities for a considered part or a product. By using AND/OR connectors and links, a network can easily be traversed and a feasible alternative process plan can be generated.

Knowledge-based representation is the second method used for representing process plans [8,39]. Here, a process plan is represented using vectors with n bits of data information. Each bit represents a machining operation and the order of bits form an operation sequence. As mentioned, each machining operation requires a set of a machine candidate, a cutting tool candidate and a TAD candidate which are used in order to perform a given machining operation. Therefore, apart from an operation vector, this representation also consists of a machine vector, a tool vector and a TAD vector.

To form a process plan, we considered that each machining feature on a part contains a certain number of operation units [32]. Operation units represent single machining operations associated with its candidate resources and can be formulated using the following expression:

(1) $${\mathrm{mo}}_{i}=\left[M_{j},\;CT_{k},\;TAD_{l}\right]$$

where ${\mathrm{mo}}_{i}$ represents a machining operation *i*, while $M_{j},\;CT_{k}$ and $TAD_{l}$ stand for a machine candidate, a cutting tool candidate and a TAD candidate for an operation *i*, respectively.

**Figure 1:**
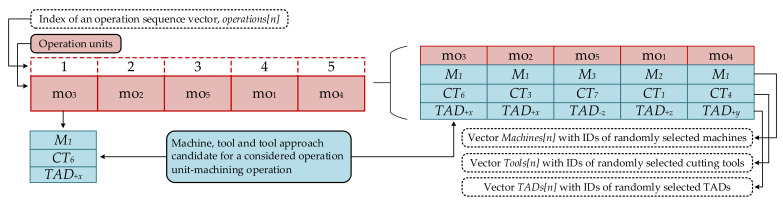
Process plan representation.

Operation units become building elements of an operation sequence that can be represented using modified knowledge-based approach similar to the one previously mentioned. Figure 1 shows a simple example of a representation of process planning problem.

In Figure 1, we have the total of 5 operation units that match the permutation of 5 numbers. They are shown in the dashed boxes in the first row of the represented process plan (left picture). Position 1 in the vector contains the operation unit 3 which further include *M*_1_—machine 1, *CT*_6_—cutting tool 6 and *TAD_+x_*—TAD “+x” as candidates for mo_3_—machining operation 3. All these machining operations form an operation sequence vector called *operations[n]* (*n* is a total number of machining operations). Candidate solutions are assigned to each machining operation and presented in the right picture. Therefore, randomly selected candidates form machine vector, cutting tool vector and TADs vector, respectively.

### 3.2. Manufacturing Relationships and Precedence Constraints

Process planning is a precondition for executing a machining process. According to manufacturing and geometric specifications of machining features, manufacturing relationships occur followed by potential interferences between certain consecutive operations. Therefore, in order to machine a feature, process planners are required to pay thorough attention to manufacturing relationships which strongly affect the feasibility of process plans. Consequently, generating operation sequence without considering manufacturing relationships may result in a situation where executing one operation may make it difficult or impossible to execute others. These relationships form precedence constraints in the PPO problem which affect the sequence of all machining operations required to machine a certain product or a part. With the emphasis on machining, two distinctive types of precedence constraints based on relationships among machining operations are hard and soft constraints [40].

Since the PPO problem considered in this study is formulated as an optimization problem, precedence constraints need to be respected in order to find feasible and optimal process plans. Generally, as the name implies, hard constraints are more stringent compared to soft constraints. They directly affect the feasibility of process plans and process planners must ensure the consistency with hard constraints. On the other hand, soft constraints have the influence solely on the quality, cost and efficiency of a feasible process plan and therefore can be violated in some cases.

**Figure 2:**
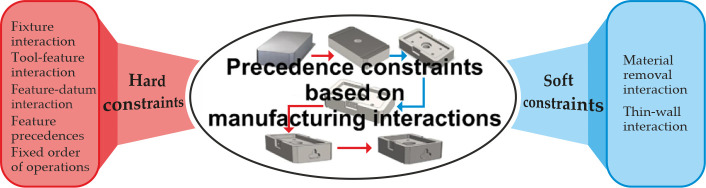
Schematic representation of precedence constraints.

According to [39,40], hard and soft constraints can be divided into several types. Figure 2 represents the schematic illustration of precedence constraints based on manufacturing relationships among features.

### 3.3. Representation and Modelling of Precedence Constraints

Precedence constraints define precedence relationships between features/machining operations whose purpose is to help in finding a feasible operation sequence. With the emphasis on hard and soft constraints, we will consider two different approaches in this research. One approach will focus only on hard precedence constraints and the other will consider both types of constraints, hard and soft. Accordingly, two different prismatic parts adopted from the literature sources are adopted to identify precedence relationships and define types of constraints based on relationships or interactions between machining operations.

The simplest way to graphically illustrate precedence relationships is with operation precedence graphs (OPGs), which are effectively proposed in [8,41]. Assuming the fact that the emphasis will be placed on two different approaches, we will adopt two different OPGs. For hard constraints only, representation will be based on classical connected OPGs (cOPGs), while for both hard and soft constraints representation of precedence relationships will be based on disconnected graphs (dOPGs).

**Figure 3:**
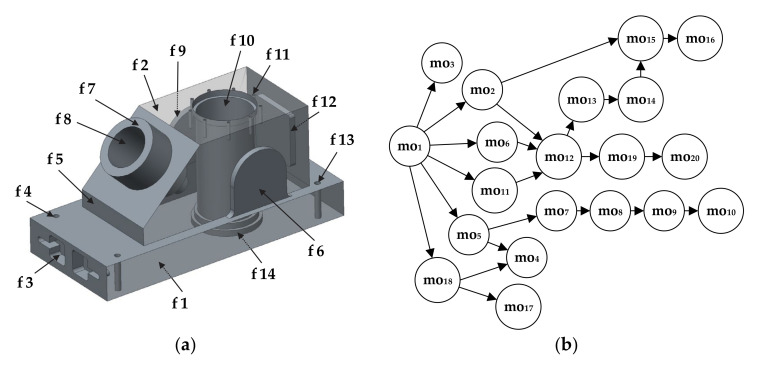
(**a**) Semi-transparent solid model and (**b**) the cOPG of the prismatic part 1.

In order to represent aforementioned constraints and relationships, we adopted the first part from Guo et al. [42]. Its 3D solid model with assigned features and operations, as well as the cOPG that depicts only hard constraints, are shown in Figure 3. According to this figure, the total number of machining operations for prismatic part 1 is 20 and can be defined as the set of machining operations [mo_1_, mo_2_, mo_3_,…, mo_20_]. Each machining operation from the cOPG is represented as a node while connections among these nodes are directed edges that show precedence relationships among them. An operation sequence that satisfies these precedence relationships is considered a feasible operation sequence.

The best way to manipulate such graphical data in a selected programming environment is by converting it to a matrix form. In that sense, we adopted the adjacency matrix [8]. The adjacency matrix is generally used to represent nodes of a graph. To convert graph information to an adjacency matrix, we used the following expression:

(2) $$Adj\_matrix=(PR_{ij})n\times n$$

where $PR_{ij}$ stands for a precedence relationship between machining operation *i* (regarding the row) and machining operation *j* (regarding the column). For hard constraints only, the $PR_{ij}$ values are binary, ones and zeroes. The first rule for this case is when $PR_{ij}=1$ and $\;PR_{ji}=0$. Here, a directed edge connects the nodes $mo_{i}$ and $mo_{j}$ pointing from ${\mathrm{mo}}_{i}$ towards ${\mathrm{mo}}_{j}$ and, therefore states that machining operation *i* has to be performed prior to machining operation *j*. Otherwise, $PR_{ij}=0$. Another rule is where $PR_{ii}=0$ for all operations from the operation set (here [mo_1_,…, mo_20_]). The expression n×n means that the adjacency matrix is a square matrix where *n* stands for the total number of machining operations.

**Figure 4:**
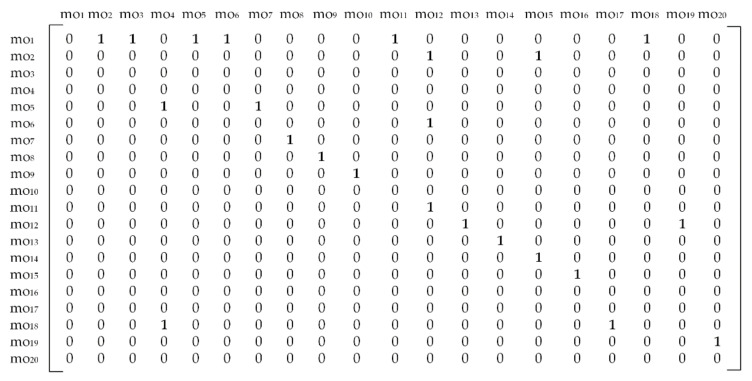
Schematic representation of precedence constraints.

It can be noticed from Figure 4, that the first row for mo_1_ regarding the machining operation 1 has six precedence relationships that equal number 1, meaning that the machining operation mo_1_ has to be performed prior to mo_2_, mo_3_, mo_5_, mo_6_, mo_11_ and mo_18_. The same rule stands for each row in the matrix.

**Figure 5:**
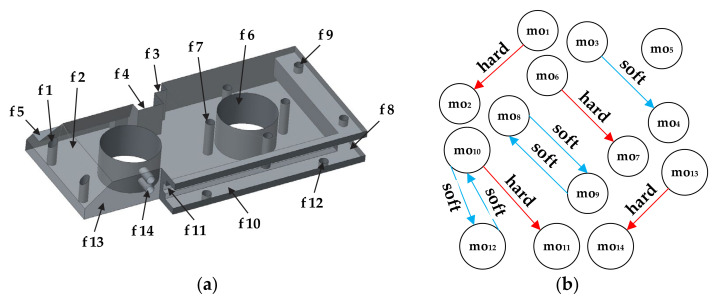
(**a**) Semi-transparent solid model and (**b**) the dOPG of the prismatic part 2.

To put an emphasis on both hard and soft constraints we included another prismatic part proposed by [3]. Three-dimensional (3D) solid model of the prismatic part 2 with assigned features and machining operations along with the corresponding dOPG is shown in Figure 5.

**Figure 6:**
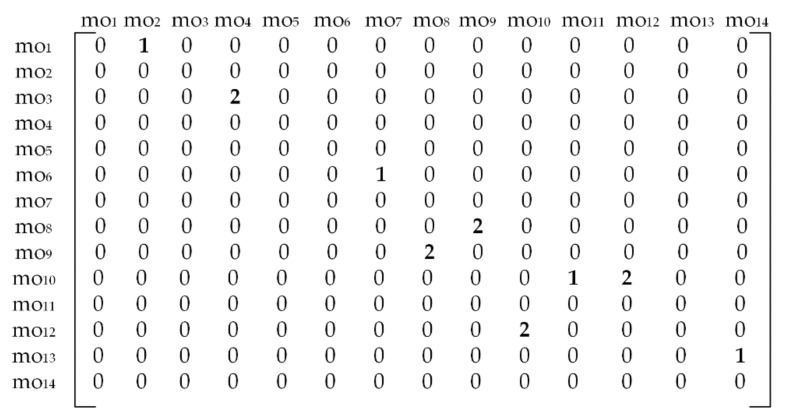
The adjacency matrix for the prismatic part 2.

Figure 5b presents the disconnected dOPG with 9 different precedence relationships among machining operations. Only node mo_5_ does not have precedence relationships with other nodes. There are 4 hard constraints and 5 soft constraints depicted with red and blue edges. Accordingly, two different numbers have to be used in order to form a reliable adjacency matrix. Herewith, number 1 stands for a hard constraint and number 2 in the matrix assumes that node ${\mathrm{mo}}_{i}$ and node ${\mathrm{mo}}_{j}$ form a soft constraint. The formulation and rules are the same as for the cOPG. The operation precedence matrix for the prismatic part 2 is given in Figure 6. Hard constraints are the ones whose violation must be avoided and therefore have total priority over soft constraints. This means that relationships among machining operations assigned with number 2 in the matrix can in some cases be neglected if they get into conflict with hard relationships assigned with number 1.

### 3.4. Mathematical Model of Process Planning

So far, the most popular evaluation criteria for the PPO problem are the minimization of the total machining time and the minimization of the total machining cost. The criterion of the total machining time has been successfully used in [9,11,20,21,25]. The machining time is composed of five time factors: machine processing time, tool processing time, transportation time (between machines), total tool change time and total setup change time. On the other side, the minimization of the total machining cost has been more frequently considered in scientific studies [4,6,8,10,11,14,15,16,24,29,31,32]. This criterion is adopted here, and consists of five cost components that are described below.

1. Total machine cost (*TMC*) represents the total cost of all machines that are used in a process plan and is calculated using the following equation:

(3) $$TMC=\sum_{i=1}^{n}MCI_{j}$$

where *j* stands for *operations[i].Machines* (i.e., *j = 1…nMach*, where *nMach* stands for total number of machines), *n* is the total number of machining operations while $MCI_{j}$ represents the machine candidate cost index for using a machine *j*, a constant cost value for a specific machine.

2. Total cutting tool cost (*TCTC*) is the total sum of cutting tool costs used in a process plan and is calculated in the following way:

(4) $$TCTC=\sum_{i=1}^{n}CTCI_{j}$$

where *j* stands for *operations[i].Tools* (*j = 1…nTools,* where *nTools* stands for total number of cutting tools), $CTCI_{j}$ stands for the cutting tool cost index for using a cutting tool *j*, a constant cost value for a specific cutting tool.

3. Total machine change cost (*TMCC*) and the number of machine changes (*NMC*) are important cost factors considered when two successive machining operations in *operations[n]* are performed on different machines. Number of machine changes is computed as:

(5) $$NMC=\sum_{i=1}^{n-1}\Omega_{1}\;\left(operations\left[i\right].Machines,\;operations\left[i+1\right].Machines\right)$$

(6) $$TMCC=\sum_{1}^{NMC}MCC$$

(7) $$\Omega_{1}\left(X,Y\right)=\left\{\begin{array}{c} 1,\;\mathrm{if}\;X\neq Y\\ 0,\;\mathrm{if}\;X=Y \end{array}\right.$$

where *MCC* represents the machine candidate change cost index while *operations[i].Machines* stands for the machine ID used for performing the machining operation *i* from *operations[n]*.

4. Total cutting tool change cost (*TCTCC*) and the number of cutting tool changes (*NCTC*) are similarly significant cost factors compared to those of machines. Cutting tool changes do not occur only in situations when same cutting tool and same machine are used for machining two successive machining operations. In other cases, tool changes are required. Accordingly, number of cutting tool changes is computed as follows:

(8) $$NCTC=\sum_{i=1}^{n-1}\Omega_{2}\left(\begin{array}{c} \Omega_{1}\;\left(\begin{array}{c} operations\left[i\right].Machines,\;\\ operations\left[i+1\right].Machines \end{array}\right),\\ \Omega_{1}\;\left(\begin{array}{c} operations\left[i\right].Tools,\;\\ operations\left[i+1\right].Tools \end{array}\right) \end{array}\right)$$

(9) $$TCTCC=\overset{NCTC}{\underset{1}{\sum }}CTCC$$

(10) $$\Omega_{2}\left(X,Y\right)=\left\{\begin{array}{c} 0,\;X=Y=0\\ 1,\;\mathrm{otherwise} \end{array}\right.$$

where *CTCC* is the cutting tool change cost index and *operations[i].Tools* stands for the cutting tool ID used for performing the operation *i* from *operations[n].*

5. Total setup cost (*TSC*), number of setup changes (*NSC*) and the number of setups (*NS*) are the cost factors considered when two successive machining operations are not performed on the same machine and using the same tool approach direction. This stands for 3-axis machines which are considered in Section 4. Firstly, the *NSC* is calculated as follows:

(11) $$SC=\sum_{i=1}^{n-1}\Omega_{2}\left(\begin{array}{c} \Omega_{1}\;\left(\begin{array}{c} operations\left[i\right].Machines,\;\\ operations\left[i+1\right].Machines \end{array}\right),\\ \Omega_{1}\;\left(\begin{array}{c} operations\left[i\right].TADs,\;\\ operations\left[i+1\right].TADs \end{array}\right) \end{array}\right)$$

The corresponding *NS* is calculated as:

(12) NS=NSC+1

Number one assumes the starting machine setup which is then added to the corresponding number of setup changes calculated by Equation (11). Lastly, the *TSC* costs are computed as:

(13) $$TSC=\sum_{1}^{NS}SCC$$

where *SCC* represent the setup change cost index and *operations[i]. TADs* stands for the TAD ID used for performing the operation *i* from *operations[n]*. Similar to tool changes, setup changes do not appear only when the same TAD and the same machine are used when performing two successive machining operations. In other cases, the change is required.

6. Additional penalty costs (*APC*) and the number of violating constraints (*NVC*) are the cost factors included in the part of our study focused on soft and hard constraints. Even if soft constraints are allowed to be violated, number of violations is subject to minimization. Firstly, the number of violated soft constraints is calculated using the following equation:

(14) $$NVC=\sum_{i=1}^{n-1}\sum_{j=i+1}^{n}\Omega_{3}\left(\begin{array}{c} operations\left[i\right],\;\\ operations\left[j\right] \end{array}\right)$$

where *operations[i]* and *operations[j]* stand for two consecutive machining operations in *operations[n]* vector. The *APC* is thereby calculated as:

(15) $$APC=\sum_{1}^{NVC}penalty$$

(16) $$\Omega_{3}\left(X,Y\right)=\left\{\begin{array}{c} 0,\;X\to Y\;\mathrm{violates}\;\mathrm{soft}\;\mathrm{constr}.\\ 1,\;X\to Y\;\mathrm{in}\;\mathrm{accordance}\;\mathrm{with}\;\mathrm{soft}\;\mathrm{constr}. \end{array}\right.$$

where *penalty* assumes the fixed penalty cost index which is applied for each violated soft constraint. In this study, the penalty cost applies to prismatic part 2 that involves both hard and soft constraints. As the conflicts between these two types of precedence constraints may occur, hard constraints become the priority. In that case, soft constraints that are numerically assigned with number 2 in Figure 6, have to be violated.

7. Total weighted machining cost (*TWMC*) sums up all the previous cost factors in the following equation:

(17) $$TWMC=w_{1}\cdot TMC+w_{2}\cdot TCTC+w_{3}\cdot TMCC+\;+w_{4}\cdot TCTCC+w_{5}\cdot \;TSC+w_{6}\cdot \;APC$$

where $w_{1}-w_{6}$ are the weight coefficients used for experimental studies. The *APC* costs are not included in Equation (17) in those studies which consider only hard precedence constraints.

8. Fitness function is maximized in order to verify the total weighted machining costs of a process plan and is defined as follows:

(18) $$f_{f}=\frac{1}{TWMC}$$

## 4. A Hybrid Grey Wolf Optimizer (HGWO) for Process Planning Optimization

### 4.1. Traditional Grey Wolf Optimizer

Grey wolf optimizer (GWO) is proposed by Mirjalili et al. [37]. It is a modern swarm-based algorithm whose inspiration derives from the social intelligence of grey wolves.

Grey wolves (lat. *Canis lupus*) are predatory animals which brings them to the top of food chain in animal world. The advantages of social life of grey wolves mostly reflect in social hunting, group care about infants and territorial defense against dangerous forces outside the pack. A strict social hierarchy among members of the pack is what brings grey wolves to the fore. From the most dominant to the most submissive wolves there are alphas, betas, deltas and omegas. According to the social dominance hierarchy, a mathematical model of GWO was designed. Three main stages of grey wolf hunting process, such as tracking, encircling and attacking a prey, are included in this model.

According to the social dominance hierarchy previously described, a mathematical model of GWO was designed. Three main stages of grey wolf hunting process, such as tracking, encircling and attacking a prey, are included in this model.

When designing the social hierarchy in the GWO, the three best solutions in a population of individuals represent alpha (*α*), beta (*β*) and delta (*δ*) wolves, respectively. All the other candidates are considered to be omegas (*ω*). The hunting (optimization) process is guided by the three fittest wolves.

The first mechanism in the GWO is encircling of prey which is defined according to the following equations:

(19) $$\vec{D}=\left|\vec{C}\cdot \vec{X_{p}}\left(it\right)-\vec{X}\left(it\right)\right|$$

(20) $$\vec{X}\left(it+1\right)=\vec{X_{p}}\left(it\right)-\vec{A}\cdot \vec{D}$$

(21) $$\vec{A}=2\vec{a}{\vec{r}}_{1}-\vec{a}$$

(22) $$\vec{C}=2{\vec{r}}_{2}$$

where *it* stands for current iteration, $\vec{A}$ and $\vec{C}$ are coefficient vectors that are defined accoding to random numbers ${\vec{r}}_{1}$ and ${\vec{r}}_{2}$ in [0,1]. $\vec{X}$ is the position vector of the prey while $\vec{X_{p}}$ is the position vector of a grey wolf. Vector $\vec{a}$ indicate a value that linearly decreases from 2 to 0.

When modelling hunting behaviour, the fittest wolves, the alpha (*α*), the beta (*β*) and the delta (*δ*) have the main role. It is assumed that these wolves respectively have better knowledge about potential location of the prey. Therefore, omega wolves (*ω*) update their positions based on the positions of the alpha (*α*), the beta (*β*) and the delta (*δ*). This process is mathematically formulated by the following equations:

(23) $${\vec{D}}_{\alpha }=\left|{\vec{C}}_{1}\cdot \vec{X_{\alpha }}-\vec{X}\right|\;\;\;{\vec{D}}_{\beta }=\left|{\vec{C}}_{2}\cdot \vec{X_{\beta }}-\vec{X}\right|\;\;\;{\vec{D}}_{\delta }=\left|{\vec{C}}_{3}\cdot \vec{X_{\delta }}-\vec{X}\right|$$

(24) $${\vec{X}}_{1}={\vec{X}}_{\alpha }-{\vec{A}}_{1}\cdot \left({\vec{D}}_{\alpha }\right)\;\;\;{\vec{X}}_{2}={\vec{X}}_{\beta }-{\vec{A}}_{2}\cdot \left({\vec{D}}_{\beta }\right)\;\;\;{\vec{X}}_{3}={\vec{X}}_{\delta }-{\vec{A}}_{3}\cdot \left({\vec{D}}_{\delta }\right)$$

(25) $$\vec{X}\left(it+1\right)=\frac{{\vec{X}}_{1}+{\vec{X}}_{2}+{\vec{X}}_{3}}{3}$$

where $\vec{X}$ represents a position of a grey wolf over a cource of iterations *it* updated according to the distances from three fittest wolves, alpha, ${\vec{D}}_{\alpha }$, beta, ${\vec{D}}_{\beta }$ and delta, ${\vec{D}}_{\delta }$.

Intensification and diversification of the GWO are maintained by the coefficient vector $\vec{A}$ whose fluctuation range depends on linear decrease of the vector $\vec{a}$. During iterations, the vector $\vec{a}$ tends to decrease the coefficient vector $\vec{A}$ which has direct impact on exploatation and exploration. If the absolute value of $\vec{A}$ is less then 1, the wolves perform an attack, i.e., converge towards the prey, and therefore local search abilities of the GWO are emphasized. On the other hand, if the absolute value of $\vec{A}$ is greater then 1, the wolves diverge from the prey and global search abilities of the GWO are used.

Search for prey, or divergence from prey, is another component of the GWO that emphasizes exploration. It is defined by the coefficient vector $\vec{C}$ which ranges from 0 to 2. Its purpose is to assign random weights to a potential prey and give wolves harder or easier way to reach it. In order to use global capacities of the GWO and avoid global optima, the value of the vector $\vec{C}$ does not decrease linearly compared to the vector $\vec{A}$.

Grey wolf optimizer has been applied to various types of optimization problems to this day. One of its main advantages is less parameter tunning since all the vectors above mentioned are based on random values. However, main drawbacks of the GWO are low convergence rate and entrapment in local optima [20]. To overcome these issues, researchers have proposed different ways to improve or enhance the traditional GWO. Ahmed et al. [43] proposed niching GWO to deal with multi-modal optimization problems. To maintain balance between exploitation and exploration and avoid premature convergence, authors incorporated best features of the PSO algorithm and a local search technique. Yue et al. [44] developed a novel hybrid algorithm based on the GWO and fireworks algorithm (FWA) in order to combine both global abilities of FWA and local abilities of GWO. Wang and Wang [45] used quantum computing principles and operations of differential evolution with grey wolf optimizer in order to improve performance when dealing with NP-complete combinatorial 0–1 knapsack problems. Martin et al. [46] proposed a novel discrete GWO algorithm which uses update rules to distinguish between exploitation and exploration stage. Jiang and Zhang [47] applied the GWO algorithm on job shop and flexible job shop scheduling problems. They embedded crossover operation and adaptive mutation method to maintain the search within discrete domain, and avoid premature convergence and local optima. Variable neighborhood search was also introduced to improve explorative capacities of the proposed GWO. Qin et al. [48] implemented hybrid discrete multi-objective GWO to solve the casting production scheduling problem where makespan, the total production cost and the total delivery delay time were used as objective functions. A strategy based on reducing job transportation time and processing time are adopted to improve initial solutions. Additionally, improved tabu search algorithm was incorporated into the GWO to improve performances of the proposed method. Premkumar et al. [49] developed a multi-objective GWO to solve the brushless direct current (BLDC) motor design problem. Firstly, the analytical model of the BLDC motor design problem that belongs to highly non-linear electromagnetic optimization problems is presented. MOGWO was verified on standard benchmark functions, and latter applied to mono and multi objective optimization of BLDC motor design problem.

In the next section, the hybrid grey wolf optimizer based on traditional GWO and GA operations is proposed for solving NP-hard process planning combinatorial problem.

### 4.2. HGWO Methodology

When designing mathematical model of the HGWO method, certain adaptations of previous Equations (19), (20), (23) and (24) have to be considered. Firstly, the distances of gray wolves from the alpha (*α*), the beta (*β*) and the delta (*δ*) wolf for the machine vector, the tool vector and the TAD vector respectively, are formulated as follows:

(26) $$\vec{D_{\alpha ,m}}=\left|{\vec{C}}_{1}\cdot \vec{X_{\alpha ,m}}-\vec{X_{m}}\right|,\;\vec{D_{\beta ,m}}=\left|{\vec{C}}_{2}\cdot \vec{X_{\beta ,m}}-\vec{X_{m}}\right|,\;\vec{D_{\delta ,m}}=\left|{\vec{C}}_{3}\cdot \vec{X_{\delta ,m}}-\vec{X_{m}}\right|$$

(27) $$\vec{D_{\alpha ,t}}=\left|{\vec{C}}_{1}\cdot \vec{X_{\alpha ,t}}-\vec{X_{t}}\right|,\;\vec{D_{\beta ,t}}=\left|{\vec{C}}_{2}\cdot \vec{X_{\beta ,t}}-\vec{X_{t}}\right|,\;\vec{D_{\delta ,t}}=\left|{\vec{C}}_{3}\cdot \vec{X_{\delta ,t}}-\vec{X_{t}}\right|$$

(28) $$\vec{D_{\alpha ,tad}}=\left|{\vec{C}}_{1}\cdot \vec{X_{\alpha ,tad}}-\vec{X_{tad}}\right|,\;\vec{D_{\beta ,tad}}=\left|{\vec{C}}_{2}\cdot \vec{X_{\beta ,tad}}-\vec{X_{tad}}\right|,\;\vec{D_{\delta ,tad}}=\left|{\vec{C}}_{3}\cdot \vec{X_{\delta ,tad}}-\vec{X_{tad}}\right|$$

where $\vec{X_{m}}$ stands for the machine position vector of a grey wolf, $\vec{X_{\alpha ,m}}$, $\vec{X_{\beta ,m}}$ and $\vec{X_{\delta ,m}}$ are the alpha (*α*), the beta (*β*) and the delta (*δ*) wolf for the machine vector; $\vec{X_{t}}$ is the tool position vector of a grey wolf, $\vec{X_{\alpha ,t}}$,$\;\vec{X_{\beta ,t}}$ and $\vec{X_{\delta ,t}}$ are the alpha (*α*), the beta (*β*) and the delta (*δ*) wolf for the tool vector, $\vec{X_{tad}}$ is the TAD position vector of a grey wolf and $\vec{X_{\alpha ,tad}}$, $\vec{X_{\beta ,tad}}$ and $\vec{X_{\delta ,tad}}$ are the alpha (*α*), the beta (*β*) and the delta (*δ*) wolf for the TAD vector.

Furthermore, the position of a gray wolf in the iteration *it + 1* is formulated on the basis of the machine vector, the tool vector and the TAD vector relative to the position vectors of the alpha (*α*), the beta (*β*) and the delta (*δ*) wolves. This can be expressed with the following expressions:

(29) $$\vec{X_{m}}\left(it+1\right)=\frac{\vec{X_{1,m}}+\vec{X_{2,m}}+\vec{X_{3,m}}}{3},\;\vec{X_{t}}\left(it+1\right)=\frac{\vec{X_{1,t}}+\vec{X_{2,t}}+\vec{X_{3,t}}}{3},\;\vec{X_{tad}}\left(it+1\right)=\frac{\vec{X_{1,tad}}+\vec{X_{2,tad}}+\vec{X_{3,tad}}}{3}$$

(30) $$\vec{X_{1,m}}=\vec{X_{\alpha ,m}}-{\vec{A}}_{1}\cdot \left(\vec{D_{\alpha ,m}}\right),\;\vec{X_{2,m}}=\vec{X_{\beta ,m}}-{\vec{A}}_{2}\cdot \left(\vec{D_{\beta ,m}}\right),\;\vec{X_{3,m}}=\vec{X_{\delta ,m}}-{\vec{A}}_{3}\cdot \left(\vec{D_{\delta ,m}}\right)$$

(31) $$\vec{X_{1,t}}=\vec{X_{\alpha ,t}}-{\vec{A}}_{1}\cdot \left(\vec{D_{\alpha ,t}}\right),\;\vec{X_{2,t}}=\vec{X_{\beta ,t}}-{\vec{A}}_{2}\cdot \left(\vec{D_{\beta ,t}}\right),\;\vec{X_{3,t}}=\vec{X_{\delta ,t}}-{\vec{A}}_{3}\cdot \left(\vec{D_{\delta ,t}}\right)$$

(32) $$\vec{X_{1,tad}}=\vec{X_{\alpha ,tad}},-{\vec{A}}_{1}\cdot \left(\vec{D_{\alpha ,tad}}\right),\;\vec{X_{2,tad}}=\vec{X_{\beta ,tad}}-{\vec{A}}_{2}\cdot \left(\vec{D_{\beta ,tad}}\right),\;\vec{X_{3,tad}}=\vec{X_{\delta ,tad}}-{\vec{A}}_{3}\cdot \left(\vec{D_{\delta ,tad}}\right)$$

where $\vec{X_{m}}\left(it+1\right)$, $\vec{X_{t}}\left(it+1\right)$ i $\vec{X_{tad}}\left(it+1\right)$ represent the updated machine, tool and TAD position vectors of a grey wolf in iteration it+1 respectively, $\vec{X_{1,m}}$, $\vec{X_{2,m}}$ and $\vec{X_{3,m}}$ are the updated machine vectors according to the distance from the alpha (*α*), the beta (*β*) and the delta (*δ*) wolf respectively, $\vec{X_{1,t}}$, $\vec{X_{2,t}}$ and $\vec{X_{3,t}}$ are the updated tool vectors according to the distance from the alpha (*α*), the beta (*β*) and the delta (*δ*) wolf respectively, and $\vec{X_{1,tad}}$, $\vec{X_{2,tad}}$ and $\vec{X_{3,tad}}$ are the updated TAD vectors according to the distance from the alpha (*α*), the beta (*β*) and the delta (*δ*) wolf respectively.

**Figure 7:**
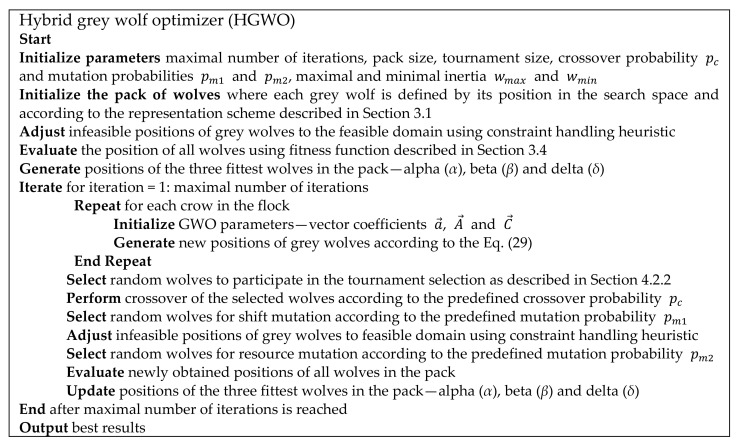
Pseudocode of the proposed HGWO.

So far, no recent discoveries have shown the proposal of the GWO for solving process planning optimization problem. In this paper, we present a novel hybrid grey wolf optimizer (HGWO) approach to address the PPO problem with various precedence constraints focusing on prismatic parts. The pseudocode of the proposed approach can be seen in Figure 7. It includes the following components: (1) knowledge-based representation of process plans (positions of grey wolves), (2) initializing population (pack of wolves), (3) applying constraint handling heuristic, (4) fitness evaluation of positions of wolves, (5) traditional GWO steps for updating positions of wolves, (6) tournament selection, (7) crossover, (8) shift mutation, (9) resource mutation, (10) fitness re-evaluation and (11) termination criteria of the HGWO.

#### 4.2.1. Initialization of Population

The first step after defining the representation of solutions is to initialize a starting population of individuals, here described as a pack of wolves. As mentioned earlier, the modified knowledge-based representation is adopted in this study. For the adopted representation of individuals, a population can be initialized. To ensure the feasibility of individuals, the search methodology has to include an appropriate approach for converting infeasible individuals to a feasible domain. Therefore, we propose two heuristic algorithms for constraint handling that will be incorporated into the proposed HGWO in order to guarantee feasible solutions. The first heuristic is adopted from [8] and utilized for dealing with hard constraints only. The other heuristic was developed in [39] to address both hard and soft constraints.

**Figure 8:**
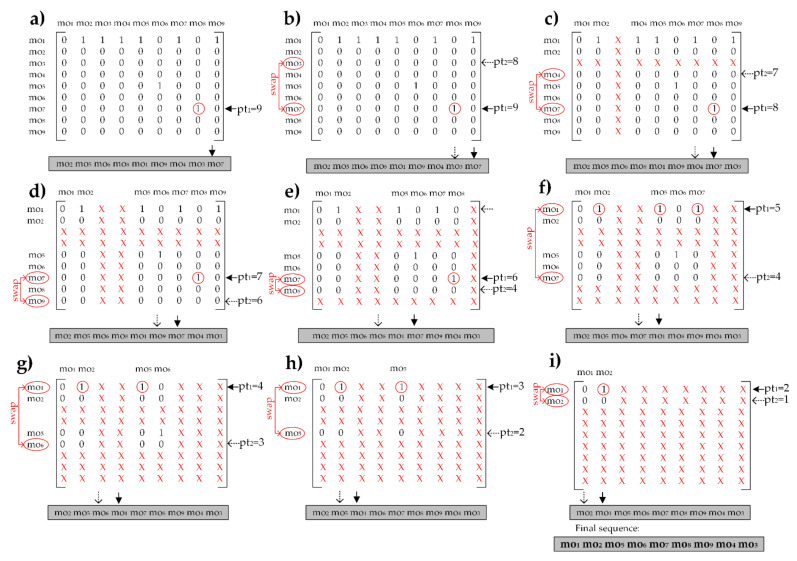
The step-by-step procedure of the constraint handling heuristic: (**a**) Select the pointer identifying the last machining operation in the sequence, mo_7_; (**b**) Swap mo_7_ and mo_3_ according to the condition in Step 5.II; (**c**) Exclude row and column of mo_3_, and select the next one, mo_4_; (**d**) Swap mo_7_ and mo_4_ according to the Step 5.II, exclude row and column of mo_4_, and select the next operation, mo_9_; (**e**) Swap positions of mo_7_ and mo_9_, exclude row and column of mo_9_, and select the next operation, mo_8_; mo_1_ is skipped according to the Step 5.II.f); (**f**) Swap positions of mo_7_ and mo_8_, exclude row and column of mo_8_, and select the next operation, mo_1_; (**g**) Swap positions of mo_7_ and mo_1_, exclude row and column of mo_7_, and select the next operations, mo_1_ and mo_6_; (**h**) Leave mo_6_ and mo_1_ as positioned, exclude row and column of mo_6_, and select the next operation, mo_5_; (**i**) mo_1_ goes before mo_5_ and before the last operation mo_2_; the final sequence is generated.

Using the information about precedence constraints and operations precedence graph let us assume that a randomly generated sequence of operations for a single individual from a population would have the following form: [mo_2_, mo_5_, mo_6_, mo_8_, mo_1_, mo_9_, mo_4_, mo_3_, mo_7_]. To complete the initialization of a process plan, randomly selected machine, cutting tool and TAD candidate are assigned to each of these machining operations to form the operation units. Here, we will focus on the operations sequence which is crucial for elimination of infeasible solutions. Using the adjacency matrix as an input information, the constraint handling heuristic for the hard constraints is developed, Figure 8. The step-by-step procedure of the constraint handling heuristic algorithm is the following:

1. A vector *operation[n]* is used for representing a single solution; its adjacency matrix is given in Equation (2);
2. A variable *pt*_1_ *(pt*_1_ *= n)* representing an index pointer of the vector *operations[n]* is generated;
3. Repeat the procedure within the for loop until *pt*_1_ *= 0*;
4. Identify the machining operation at the point *pt*_1_ *= n*;
5. For the identified machining operation calculate the sum of all number values in a corresponding row of the adjacency matrix $\sum_{i=1}^{n}(PR_{ij})$; Then, check the conditions:
  - If $\sum_{i=1}^{n}(PR_{ij})=0$
    a) Leave the machining operation at the position *pt*_1_;
    b) Exclude the row and the column of the *Adj_matrix* that match the value of the machining operation at the position *pt*_1_; instead of removing the row and the column, assign “not a number”;
    c) Initialize *n = n − 1* and move pointer *pt*_1_ one place left;
  - If $\sum_{i=1}^{n}(PR_{ij})\neq 0$
    (a) Initialize *pt*_2_ *= pt*_1_ *− 1*;
    (b) Identify the machining operation at the position *pt*_2_;
    (c) Calculate the sum $\sum_{i=1}^{n}(PR_{ij})$ of all number values in the corresponding row that matches the identified machining operation at the position *pt*_2_;
    (d) Repeat the following steps while *pt*_2_ *> 0*;
    (e) If $\sum_{i=1}^{n}(PR_{ij})=0$
      ▪ Swap the machining operation at the position *pt*_1_ with the machining operation at the position *pt*_2_;
      ▪ Remove the row and the column of the corresponding *Adj_matrix* which match the value of the machining operation at the position *pt*_2_; assign “not a number”;
      ▪ Break the loop and move to the step (3);
    (f) If $\sum_{i=1}^{n}(PR_{ij})\neq 0$
      ▪ Initialize *pt*_2_ *= pt*_2_ *− 1*;
      ▪ Move to the step (d);
6. Return to the step (3) and reduce the pointer *pt*_1_ *= pt*_1_ *− 1*;
7. Reset the *Adj_matrix* and apply all the steps for the next individual.

The step-by step constraint handling procedure begins with the adjacency matrix given in Figure 8a. The procedure is based on identifying index pointers *pt*_1_ and modifying the sequence within the for loop. The first selected pointer *pt*_1_ = *n* = 9 denotes the last machining operation in the matrix, mo_7_. The sum of all values in the row 9 meets the second condition in the Step 5 meaning that $\sum_{i=1}^{n}(PR_{ij})\neq 0$. Then, the Step 5.II.a) starts with initializing the second pointer *pt*_2_ *= pt*_1_ *− 1 =* *8* that denotes mo_3_, Figure 8b. The same condition is checked and since the sum $\sum_{i=1}^{n}(PR_{ij})$ equals zero, the operations mo_7_ and mo_3_ are swapped and the row and the column of the mo_3_ are assigned with the non-numbering value. Operation mo_3_ than takes the final position 8 in the sequence, Figure 8c. The procedure continues with the Step 6 that reduces the pointer *pt*_1_ to 8 and repeats from the Step 5. Pointers *pt*_1_ = 8 and *pt*_2_ *= 7* denote the operations mo_7_ and mo_4_, Figure 8c. After checking conditions in Step 5.II, the row and the column of mo_4_ are assigned with the non-numbering value and removed from the following steps, Figure 8d. From Figure 8d to Figure 8i, the handling procedure continues to remove the rows and the columns of machining operations for which the sum $\sum_{i=1}^{n}(PR_{ij})$ equals zero. The sequence of operations is modified accordingly. Figure 8i shows that the operation mo_5_ has to swap place with mo_1_ in order to complete the procedure of generating a feasible machining sequence.

The second constraint handling heuristic is adopted from [3]. Here, we will shortly focus on the most significant steps of this heuristic. In order to handle both hard and soft constraints, firstly, so called “linked list” is created. This list includes machining operations that form hard constraints, therefore leaving the positions of other operations unchanged. Herewith, a heuristic process is imposed on the linked list to ensure its consistency with the hard constraints. Such an approach is adopted for the part 2 shown in Figure 5. Afterwards, the next step of this constraint handling algorithm is to include the additional penalty costs for violated soft constraints in the objective function model, Equations (15) and (16). Considering the fact that soft constraints are not manipulated using the linked list, certain soft constraints may be subject to violation. In this step, soft constraints can be compromised and violated to achieve the minimal total weighted machining cost, Equation (17).

#### 4.2.2. Genetic Components of the HGWO Approach

Genetic algorithms are techniques based on natural evolution of species that mimic the viewpoint of modern genetics, so-called “survival of the fittest”. Apart from being fitness-oriented and belonging to the group of population-based optimization algorithms, their unique characterization is varying operations that mimic genetic gene changes and enable population individuals to evolve. These strategies are selection, crossover and mutation.

The adopted selection strategy for the HGWO algorithm is the tournament selection which belongs to the group of proportionate-based selection schemes. This strategy is based on selecting a number of individuals (tournament size) from the population that will participate in the so called “tournament”. The individual with the highest fitness value is considered as the fittest individual and therefore the winner of the tournament. The process of selecting individuals lasts until a new generation of individuals is created.

Crossover or recombination is the next strategy employed to recombine two selected parent individuals in order to obtain better offsprings. In this paper, a modified one-point crossover strategy is adopted to improve exploration capabilities of the proposed approach. Using the predefined crossover probability $p_{c}$, the steps of this recombination procedure are the following:

- Two wolf candidates selected from the tournaments are randomly chosen to be parent wolves;
- By generating a random crossover point, two parent wolves are divided into two sections to create two child wolves;
- Left section of the child wolf 1 is formed by copying left section of the parent wolf 1. Then, the right section of the child wolf 1 is formed in two parts. The first part is to find the remaining machining operations in the parent wolf 2 and copy them in the current order to the right section of the child wolf 1. Afterwards, the machine, cutting tool and TAD candidate are copied from these machining operations in the parent wolf 2 to the remaining machining operations in right section of the child wolf 1;
- The identical, but inverse procedure using left section of the parent wolf 2 and remaining machining operations from the parent wolf 1 is performed to produce the child wolf 2.

To improve exploration capabilities of the HGWO and avoid premature convergence, two appropriate mutation strategies are introduced. Here, the shift mutation and resource mutation strategy are used. The first strategy, takes two random genes (operation units) of a randomly selected wolf from the population (pack) and exchanges them. This procedure impacts the feasibility of the mutated position of the wolf.

The second mutation strategy called “resource mutation” does minor changes to a machine, cutting tool and TAD candidate, respectively. The steps of the resource mutation strategy (example for a machine vector) are the following:

- Randomly select a crow candidate for resource mutation;
- Randomly select the mutation point, i.e., machining operation that matches certain position in the vector;
- Check the availability of machines in the machine candidate list for the selected machining operation;
- Randomly select one of the available machines as the current machine;
- Identify all other machining operations that have the current machine in their machine candidate list;
- Assign the same alternative machine candidate as the current machine for remaining machining operations;
- Repeat the same steps for the CT vector and TAD vector using CT candidate list and TAD candidate list respectively.

Besides the above described genetic strategies within the HGWO approach, we also included inertia weight coefficient in order to achieve additional control of exploration and exploitation. The inertia is decreased linearly thereby emphasizing global exploration ability in the initial stages of the search process and moving towards local exploitation abilities in the latter stages. Convergence curves in Chapter 5 graphically show diversification of the search (primarily HGWO and HybGA curves) in the initial stages meaning that the exploration is at hand, whereas the latter stages clearly show less diversified search where local abilities of metaheuristics are more used. In that sense, the mathematical Equations (25)–(27) defined in the Section 4.2 will have the following form:

(33) $$\vec{D_{\alpha ,m}}=\left|{\vec{C}}_{1}\cdot \vec{X_{\alpha ,m}}-w\cdot \vec{X_{m}}\right|,\;\vec{D_{\beta ,m}}=\left|{\vec{C}}_{2}\cdot \vec{X_{\beta ,m}}-w\cdot \vec{X_{m}}\right|,\;\vec{D_{\delta ,m}}=\left|{\vec{C}}_{3}\cdot \vec{X_{\delta ,m}}-w\cdot \vec{X_{m}}\right|$$

(34) $$\vec{D_{\alpha ,t}}=\left|{\vec{C}}_{1}\cdot \vec{X_{\alpha ,t}}-w\cdot \vec{X_{t}}\right|,\;\vec{D_{\beta ,t}}=\left|{\vec{C}}_{2}\cdot \vec{X_{\beta ,t}}-w\cdot \vec{X_{t}}\right|,\;\vec{D_{\delta ,t}}=\left|{\vec{C}}_{3}\cdot \vec{X_{\delta ,t}}-w\cdot \vec{X_{t}}\right|$$

(35) $$\vec{D_{\alpha ,tad}}=\left|{\vec{C}}_{1}\cdot \vec{X_{\alpha ,tad}}-w\cdot \vec{X_{tad}}\right|,\;\vec{D_{\beta ,tad}}=\left|{\vec{C}}_{2}\cdot \vec{X_{\beta ,tad}}-w\cdot \vec{X_{tad}}\right|,\;\vec{D_{\delta ,tad}}=\left|{\vec{C}}_{3}\cdot \vec{X_{\delta ,tad}}-w\cdot \vec{X_{tad}}\right|$$

where w represents inertia weight that is decreased linearly using the next equation:

(36) $$w=\left(\left(w_{max}-w_{min}\right)/MaxIt\right)\cdot it$$

where $w_{max}$ and $w_{min}$ are maximal and minimal weight, MaxIt is the maximal number of iterations and it is the current iteration.

## 5. Experimental Studies and Results

**Figure 9:**
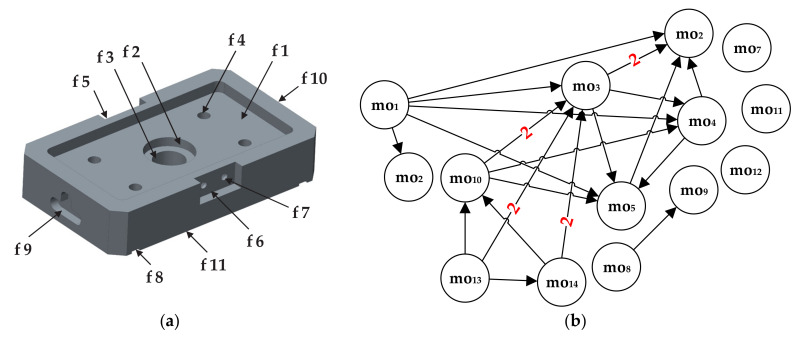
(**a**) Semi-transparent solid model and (**b**) dOPG of prismatic part 3.

**Figure 10:**
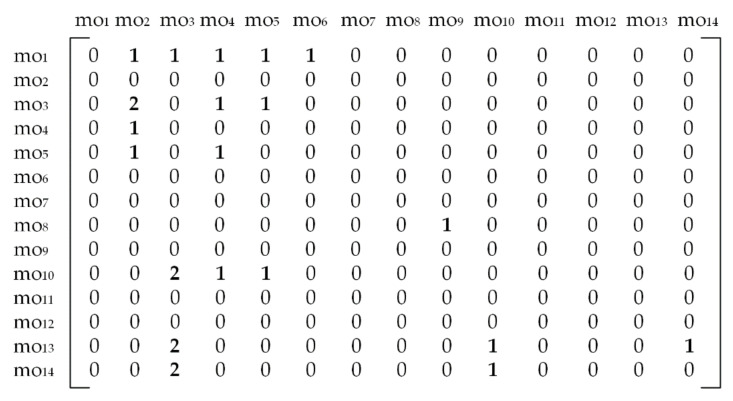
The adjacency matrix for prismatic part 3.

The proposed HGWO algorithm was programmed in MATLAB environment and executed on a Windows 10 operating system by a 1.99 GHz Intel i7 processor and 8 GB RAM computer. Its performance was verified on three experimental studies that consider three different prismatic parts. The first experiment employed the prismatic part 1 taken from [42]. The 3D solid model with associated features and its cOPG are represented in Figure 3. The second experiment for testing the HECSA algorithm is the prismatic part 2 taken from [3]. Its model representation and dOPG are presented in Figure 5. The prismatic part 3 in the third experiment is adopted from [6]. Figure 9 and Figure 10 depict the semi-transparent 3D model with its dOPG and adjacency matrix, respectively. Compared to the first and the second experimental study which considered prismatic parts with hard, and both hard and soft constraints respectively, precedence relationships in this model require certain operations to be performed in the same setup. In that sense, number 2 is added to the dOPG (e.g., mo_10_ precedes mo_3_ and both should be performed in the same setup). The prismatic part 3 is modeled for one condition in which all resources are available.

The detailed information about features and candidates for machines, cutting tools and TADs for prismatic part 1 is given in Table 1. Precedence relationships among machining features for prismatic part 1 are presented in Table 2. Similarly, information about features and resource candidates, as well as information about precedence relationships for prismatic part 2 are given in Table 3 and Table 4, respectively. Lastly, information about features and resource candidates for prismatic part 3 are shown in Table 5. Available machines and cutting tools as well as the machining cost information about three prismatic parts are given in Table 6 and Table 7, respectively.

Parameter tuning may be one of the most time-consuming challenges when testing the performances of metaheuristic algorithms. In this paper, we performed several manual tests of input parameters of the HGWO and finally we adopted the following: pack size: N=100, maximal number of iterations: MaxIter=700, tournament size: TourSize=5, crossover probability: $p_{c}=0.8$, shift mutation probability $p_{m1}=0.4$, resource mutation probability: $p_{m2}=0.6$ and maximal and minimal inertia weights are $w_{max}=1.2$ and $w_{min}=0.2$. The adopted optimization objective is to minimize the total weighted machining cost defined in Section 3.4. The output results included minimum and maximum results achieved in 20 runs, and mean values of the obtained results.

The optimal process plans for minimal machining cost and three conditions concerning prismatic part 1 are shown in Table 8. In the first condition, all resources are available. The second condition excludes costs for cutting tools and cutting tool changes. The third condition is the same as the second, with unavailability of machine 2 and cutting tool 8.

To perform detailed comparative analysis, results were compared with different approaches in the literature, HGASA by Li et al. [5], PSO by Guo et al. [42], HybGA by Huang et al. [8], HBMO by Wen et al. [24], ACO by Wang et al. [15], TSACO by Wang et al. [16], cPSO by Petrović et al. [20], mACO by Hu et al. [17], ESGA by Su et al. [11], IWD by Gao et al. [32], TS, SA, GA and ACO by Liu et al. [14] and GA by Kafashi [6]. Numerical information about the comparative studies for three prismatic parts is given in Table 9.

Firstly, we will discuss the results for the prismatic part 1. For the first condition where all resources are available, the HGWO obtained the minimal TWMC of 2470 cost units in 20 runs. The total of six results have values bellow 2500 cost units. According to Table 9, the minimal TWMC of 2470 is one of the best results compared to the results obtained by other algorithms. Only the approach reported in [15] achieved better minimum. The average cost obtained by the HGWO is better than the average cost achieved by cPSO [20], PSO [42], and mACO [17]. However, the maximal value of 2805 shows a lack of consistency in achieved results compared to other metaheuristics. Considering the second condition where CT cost and CT change cost are not included, the TWMC of 1990 cost units appears to be the best minimal result. As shown in Table 9, HGWO outperforms all other metaheuristic approaches in terms of the minimal TWMC and the mean TWMC. On the other hand, the achieved maximal value was better compared to ACO [15], TSACO [16] and IWD [32] approaches. Lastly, the HGWO showed best performances for the third condition in which machine *M*_2_ and cutting tool *CT*_8_ are not available. Best minimal TWMC of 2490 cost units is found. Additionally, regarding the mean and the maximal values of the TWMC, the HGWO demonstrated much better consistency and effectiveness than the considered modified and hybrid approaches.

**Figure 11:**
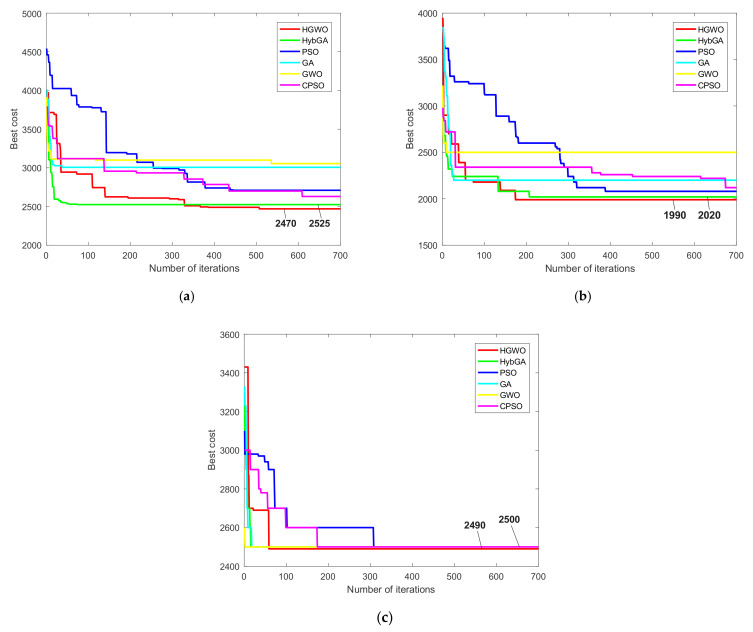
Convergence curves for the prismatic part 1: (**a**) 1st condition; (**b**) 2nd condition; (**c**) 3rd condition.

Furthermore, in Figure 11 we presented convergence curves for prismatic part 1. As can be seen, convergence of six different metaheuristic approaches is compared considering three optimization conditions. Convergence of the proposed HGWO is compared with the convergence of three traditional algorithms, PSO [42], GA [14] and GWO, as well as the hybrid and the modified metaheuristics, HybGA [8] and cPSO [20]. From the information in Figure 11a, considering the first condition, GWO, GA and HybGA showed much faster converegence compared to other algorithms. However, the GWO and GA converged towards local optima, whereas the HybGA succeded in finding much better solution. Although the HGWO showed much slower convergence than the HybGA for example, it converged towards the minimal value of 2470. This value may not be considered the global optimum, since the slightly better result in the literature has been reported so far. It could be safely argued that the HGWO converged towards the near-optimal solution. From Figure 11b and the second condition of the first study, it can be noticed that the convergence of the HGWO is similar to the one of the HybGA. After approximately 200 iterations, these approaches converged towards the optimal and near-optimal result. PSO, like CPSO, converged towards the good solutions, but with much slower convergence. GA and GWO got trapped in the local optima in the early stages. Lastly, the convergence curves for the third condition are given in Figure 11c. It can be seen that all algorithms in this analysis converged towards best solutions. The reason lies in the fact that the third condition excludes certain solution candidates as well as cost assigned with them. HGWO showed slightly slower convergence than GA, GWO and HybGA, but, on the other side, succeded in finding the new global optimum of 2490 cost units for TWMC. This result showed the effectiveness of the HGWO for the third condition.

Best process plans for two conditions for prismatic part 2 are given in Table 10. After adopting similar parameters as in the previous study, we compared the results with six different approaches from the literature [8,14,20]. The comparative results are shown in Table 9. The proposed HGWO performed well by achieving optimal TWMC of 1328 cost units eight times under the first condition. The mean value was slightly greater compared to the same result achieved by TS and ACO, but much better compared to other algorithms. Similar conclusion can be made for the maximal TWMC where the HGWO showed better result compared to the HybGA, TS, SA, GA and CPSO. Under the second condition, the HGWO found the minimal TWMC of 1170 cost units which appeared 19 times in 20 runs. Besides the ACO approach, the HGWO achieved the best maximal and the best mean results compared to all other algorithms.

**Figure 12:**
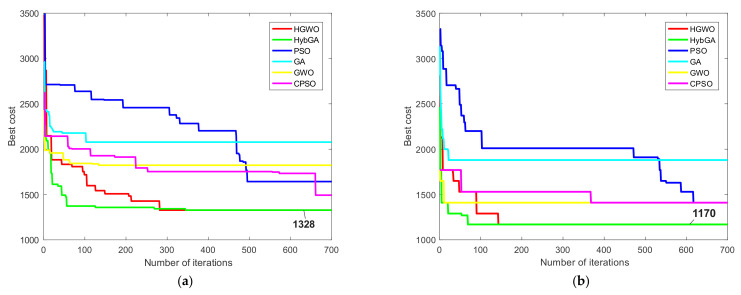
Convergence curves for the prismatic part 2: (**a**) 1st condition; (**b**) 2nd condition.

Figure 12 presents the convergence curves for two optimization conditions of the second experimental study concerning prismatic part 2. The same six metaheuristics are considered in the convergence analysis. As shown in Figure 12a for the first condition, the HGWO and the HybGA show similar performances, with the HGWO showing slower convergence compared to the hybrid metaheuristic algorithm. Both approaches converged towards the global TWMC of 1328 cost units. GA and GWO got trapped in the local optima in early stages, whereas the CPSO and the PSO showed progress in converging towards good solutions but not as good as the ones found by the HGWO and the HybGA. Similar performances can be seen for the second condition shown in Figure 12b. Since the second condition excludes certain alternatives and costs, the HGWO showed faster convergence than for the previous condition. Like the HybGA, HGWO converged towards the global TWMC of 1170 cost units. It took HGWO approximately 150th iterations to converge towards the global optimum compared to the HybGA which was faster to some extent. However, compared to the traditional GA, GWO, PSO, as well as the CPSO, the HGWO showed more superior performance for the second condition.

The optimal process plans for prismatic part 3 are obtained and presented in Table 11. The same input parameters are used for this experimental study. Table 9 shows the comparative results for the minimal, the maximal and the mean TWMC. In 20 runs, the HGWO obtained the TWMC below 1900 cost units eight times in total, and bellow 2000 cost units eleven times. The minimal TWMC of 1815 cost units is better than the minimal values obtained by the GA, PSO, as well as the HGASA. The HGWO outperforms these algorithms in terms of the best maximal and the best mean results.

**Figure 13:**
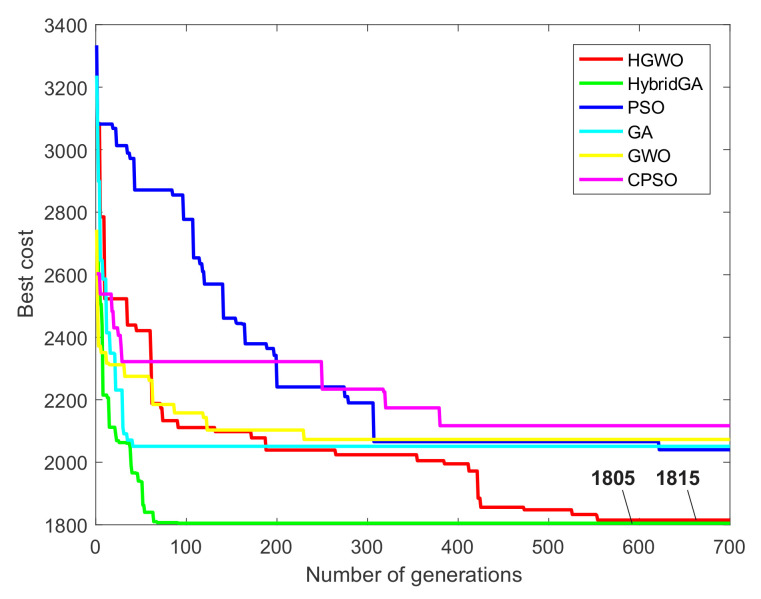
Convergence curve for the prismatic part 3.

Figure 13 shows the convergence curves for the third experimental study. It can be noticed that the HybGA again shows the best convergence rate and by far outperforms other algorithms in this regard. As far as the HGWO is concerned, it shows much slower convergence compared to the first and the second experimental study. The HGWO obtained the TWMC of 1815 cost units after 500 iterations. Despite the slow convergence rate, the proposed HGWO algorithm achieved near-optimal solution for this prismatic part. Traditional GWO and especially GA also showed good convergence rates but they got trapped in the local optima. CPSO and PSO performances could not match the performances of the previous algorithms.

## 6. Time Efficiency of the HGWO

According to the obtained experimental results, the HGWO approach showed very good effectiveness and flexibility by finding optimal and near-optimal solutions of the process planning optimization problem. From the perspective of the solutions quality, the comparative results in Table 9 show that the HGWO obtained the near-optimal TWMC value for the first condition, and the new optimal values for the second and the third condition of the first experimental study. In terms of the consistency, the HGWO showed good mean values for all three conditions surpassing the mean results of several algorithms reported in the literature. The optimal TWMC cost values are obtained for both conditions of the second experimental study with much better consistency that is reflected in very good mean results. In the third experimental study, the HGWO approach obtained near-optimal results of TWMC with good consistency where mean results surpass those results obtained by traditional algorithms.

Although the proposed HGWO approach demonstrated flexibility and effectiveness in terms of the solution qualities, there are certain limitations regarding its computational time efficiency. Due to the fact a small number of researchers included computational times in their studies, a detailed comparative analysis could not be performed in this regard. In Table 12 we presented the mean computational times of six metaheuristic algorithms whose convergence curves were shown in Figure 11, Figure 12 and Figure 13 for three experimental studies, respectively. They are expressed in seconds required to complete a single run, and the results in Table 12 are the mean computational times of 20 runs. From the perspective of the HGWO efficiency, better mean computational times are achieved against traditional PSO and modified cPSO approach. On the other side, they exceed the mean computational times of three algorithms, GWO, GA and HybGA. Similar values for the HGWO and the PSO are obtained for the prismatic part 2. Although some authors suggest less amount of time to be reasonable for large-sized NP-hard problems [50], we may argue that approximately 1 to 1.5 min of the running time is a reasonably well amount of time needed to solve different instances of an NP-hard problem such as the PPO. GA and GWO as traditional algorithms obviously show the lowest computational times in all studies, since the modified and hybrid algorithms require more time to perform additional operations. One of the reasons for lower efficiency of the HGWO compared to the HybGA is the difference in input parameters, where HGWO considered larger probabilities for crossover and mutation strategies, as well as larger tournament size, compared to HybGA. According to the convergence curves in Figure 11, Figure 12 and Figure 13, the convergence rate of the HGWO is slower in the first stages of the search process, especially for the first conditions of prismatic parts 1 and 2, as well as prismatic part 3. For example, curve in Figure 11a shows slower rate of the HGWO in the first 300 iterations, meaning that the probabilities of crossover and mutation are much higher than in the latter iterations, which leads to the increase in computational time of the HGWO.

Time efficiency of the HGWO can also be evaluated by the function execution times that are called in the programming environment. Since the MATLAB was used to implement the HGWO, we provide the execution times of several functions coded in MATLAB which are presented in Table 13. Apart from the functions that consider calling six different algorithms considered in experimental studies, fitness evaluation and constraint handling functions are also presented. Two time values, total and self-time are shown. Total time represents the total time spent in a function including all child functions, while the self-time means the total time spent in a function without any time spent in child functions. All time values in Table 13 are obtained after 20 runs and consider only the first experimental study. It can be noticed that calling the HGWO algorithm requires more time compared to almost all other algorithms except the CPSO. The big difference in total and self-time means that the HGWO requires calling many child functions, including fitness evaluation and constraint handling algorithm.

According to the computational times and function execution times, we can argue that the HGWO demonstrated good time efficiency. However, this can also be considered as the main disadvantage of the proposed approach since the comparative results clearly show that the HGWO falls behind other algorithms. The fact that the constraint handling heuristic, the fitness evaluation, the tournament selection, the crossover, the shift mutation, and the resource mutation represent a separate MATLAB functions that the HGWO has to call in order to perform a single iteration, the time efficiency should be treated with enough attention. With effectiveness and flexibility as the main advantages, and time efficiency as the main disadvantage of the HGWO approach, potential improvements in terms of time efficiency and convergence rate are beyond the scope of this paper and may be a subject for future research studies.

## 7. Conclusions

A hybrid grey wolf optimizer is proposed to solve the NP-hard process planning optimization problem based on precedence constraints. The problem considers operation sequencing task along with the optimal selection of appropriate machines, cutting tools and tool approach directions that define a process plan. Modern knowledge-based representation scheme is used to represent process plans in a population of alternatives and the operation precedence graph approach with adjacency matrices is adopted to deal with precedence relationships among machining features. According to these relationships, precedence constraints are defined. To deal with the precedence constraint in an appropriate manner, we adopted constraint handling heuristic algorithm to deal with hard constraints, as well as both hard and soft constraints. In that sense, the feasibility of process plans was ensured.

To improve the performance of traditional grey wolf optimizer, the strategies of genetic algorithm were adopted. Tournament selection is utilized for selecting fittest individuals and classical two-point crossover strategy is applied on selected individuals to generate wolf offspring. Shift and resource mutation strategies preform minor changes to vector candidates and complete the evolutionary steps of the HGWO approach. To additionally achieve balance between exploration and exploitation inertia weight coefficients from the PSO are added to the mathematical model of the HGWO approach.

For the evaluation of process plans, traditional total weighted machining cost was adopted as an optimization criterion. The detailed mathematical model is presented and the criterion was formed for different conditions covered in three experimental studies which consider three prismatic parts proposed in the literature.

After manually determining input parameters of the proposed HGWO approach, popular results obtained by traditional and modern approaches were used to perform the detailed comparative analysis. Three experimental studies are conducted to test the effectiveness and efficiency of the HGWO approach. The detailed representation of optimal process plans and comparative results including the minimal, the maximal and the mean TWMC for 20 algorithm runs were given. The HGWO approach demonstrated flexibility and effectiveness by solving the process planning optimization problem and finding the optimal and the near-optimal results.

Although effectiveness and flexibility showed as the main advantages of the HGWO, time efficiency of the proposed algorithm showed certain limitations. The HGWO succeeded in finding optimal and near-optimal process plans in a reasonable time period, but the comparative analysis of computational times, as well as function execution times in MATLAB programming environment, confirm good efficiency of the HGWO. However, the HGWO falls behind certain hybrid and traditional algorithms in this regard.

One of the directions for future research may be focused on improving efficiency and convergence of the HGWO, primarily through consideration of different genetic strategies. Additionally, optimization of input parameters has already been reported in some studies and should be taken into account in order to further improve performances of the HGWO. Possible contribution to the area of process planning optimization can be directed towards the optimization of integrated process planning and scheduling. Likewise, the recent advances in the field of Industry 4.0 may consider the implementation of the HGWO as a cloud service within the Smart factory.

## Figures and Tables

**Table 1:** Information about features and resource candidates for prismatic part 1.

Feat.	Feature Definition	Machining Operations	Machines	Cutting Tools	TADs
f1	Planar surface	Milling (mo_1_)	*M*_2_ *,M*_3_	*CT*_6_ *,CT*_7_ *,CT*_8_	*TAD_+z_*
f2	Planar surface	Milling (mo_2_)	*M*_2_ *,M*_3_	*CT*_6_ *,CT*_7_ *,CT*_8_	*TAD_−z_*
f3	Two pockets arranged as a replicated feature	Milling (mo_3_)	*M*_2_ *,M*_3_	*CT*_6_ *,CT*_7_ *,CT*_8_	*TAD_+x_*
f4	Four holes arranged as a replicated feature	Drilling (mo_4_)	*M*_1_ *,M*_2_ *,M*_3_	*CT*_2_	*TAD_+z_, TAD_−z_*
f5	A step	Milling (mo_5_)	*M*_2_ *,M*_3_	*CT*_6_ *,CT*_7_	*TAD_+x_, TAD_−z_*
f6	A rib	Milling (mo_6_)	*M*_2_ *,M*_3_	*CT*_7_ *,CT*_8_	*TAD_+y_, TAD_−z_*
f7	A boss	Milling (mo_7_)	*M*_2_ *,M*_3_	*CT*_7_ *,CT*_8_	*TAD_−a_*
f8	A compound hole	Drilling (mo_8_)	*M*_1_ *,M*_2_ *,M*_3_	*CT*_2_ *,CT*_3_ *,CT*_4_	*TAD_−a_*
		Reaming (mo_9_)	*M*_1_ *,M*_2_ *,M*_3_	*CT*_9_	
		Boring (mo_10_)	*M*_2_ *,M*_3_	*CT*_10_	
f9	A rib	Milling (mo_11_)	*M*_2_ *,M*_3_	*CT*_7_ *,CT*_8_	*TAD_−y_, TAD_−z_*
f10	A compound hole	Drilling (mo_12_)	*M*_1_ *,M*_2_ *,M*_3_	*CT*_2_ *,CT*_3_ *,CT*_4_	*TAD_−z_*
		Reaming (mo_13_)	*M*_1_ *,M*_2_ *,M*_3_	*CT*_9_	
		Boring (mo_14_)	*M*_3_ *,M*_4_	*CT*_10_	
f11	Nine holes arranged as a replicated feature	Drilling (mo_15_)	*M*_1_ *,M*_2_ *,M*_3_	*CT*_1_	*TAD_−z_*
		Tapping (mo_16_)	*M*_1_ *,M*_2_ *,M*_3_	*CT*_5_	
f12	A pocket	Milling (mo_17_)	*M*_2_ *,M*_3_	*CT*_7_ *,CT*_8_	*TAD_−x_*
f13	A step	Milling (mo_18_)	*M*_2_ *,M*_3_	*CT*_6_ *, CT*_7_	*TAD_−x_, TAD_−z_*
f14	A compound hole	Reaming (mo_19_)	*M*_1_ *,M*_2_ *,M*_3_	*CT*_9_	*TAD_+z_*
		Boring (mo_20_)	*M*_3_ *,M*_4_	*CT*_10_	

**Table 2:** Precedence relationships for prismatic part 1.

Features	Machining Operations	Precedence Constraint Description
f1	Milling (mo_1_)	f1 (mo_1_) is the datum and supporting face for the part; therefore it is machined before all features and operations
f2	Milling (mo_2_)	f2 (mo_2_) is before f10 (mo_12_, mo_13_, mo_14_) and f11 (mo_15_, mo_16_) for the material removal interactions
f3	Milling (mo_3_)	
f4	Drilling (mo_4_)	
f5	Milling (mo_5_)	f5 (mo_5_) is before f4 (mo_4_) and f7 (mo_7_) for the datum interactions
f6	Milling (mo_6_)	f6 (mo_6_) is before f10 (mo_12_, mo_13_, mo_14_) for the datum interaction
f7	Milling (mo_7_)	f7 (mo_7_) is before f8 (mo_8_, mo_9_, mo_10_) for the datum interactions
f8	Drilling (mo_8_)	mo_8_ is before (mo_9_ and mo_10_); mo_9_ is before mo_10_ for the fixed order of machining operations
	Reaming (mo_9_)	
	Boring (mo_10_)	
f9	Milling (mo_11_)	f9 (mo_11_) is before f10 (mo_12_, mo_13_, mo_14_) for the datum interaction
f10	Drilling (mo_12_)	mo_12_ is before (mo_13_ and mo_14_); mo_13_ is before mo_14_ for the fixed order of machining operations; f10 (mo_12_, mo_13_, mo_14_) is before f11 (mo_15_, mo_16_) and mo_12_ of f10 is before f14 (mo_19_, mo_20_) for the datum interaction
	Reaming (mo_13_)	
	Boring (mo_14_)	
f11	Drilling (mo_15_)	mo_15_ is before mo_16_ for the fixed order of operations
	Tapping (mo_16_)	
f12	Milling (mo_17_)	
f13	Milling (mo_18_)	f13 (mo_18_) is before f4 (mo_4_) and f12 (mo_17_) for the material removal interaction
f14	Reaming (mo_19_)	mo_19_ is before mo_20_ for the fixed order of machining operations
	Boring (mo_20_)	

**Table 3:** Information about features and resource candidates for prismatic part 2.

Feat.	Feature Definition	Machining Operations	Machines	Cutting Tools	TADs
f1	Two holes as a replicated feature	Drilling (mo_1_)	*M*_1_ *,M*_2_ *,M*_3_	*CT*_1_	*TAD_+z_, TAD_−z_*
f2	A chamfer	Drilling (mo_2_)	*M*_2_ *,M*_3_	*CT*_8_	*TAD_−x_, TAD_+y_, TAD_−y_, TAD_−z_*
f3	A slot	Milling (mo_3_)	*M*_2_ *,M*_3_	*CT*_5_ *,CT*_6_	*TAD_+y_*
f4	A slot	Milling (mo_4_)	*M*_2_	*CT*_5_ *,CT*_6_	*TAD_+y_*
f5	A step	Milling (mo_5_)	*M*_2_ *,M*_3_	*CT*_5_ *,CT*_6_	*TAD_+y_, TAD_−z_*
f6	Two holes as a replicated feature	Drilling (mo_6_)	*M*_1_ *,M*_2_ *,M*_3_	*CT*_2_	*TAD_+z_, TAD_−z_*
f7	Four holes as a replicated feature	Drilling (mo_7_)	*M*_1_ *,M*_2_ *,M*_3_	*CT*_1_	*TAD_+z_, TAD_−z_*
f8	A slot	Milling (mo_8_)	*M*_2_ *,M*_3_	*CT*_5_ *,CT*_6_	*TAD_+x_*
f9	Two holes as a replicated feature	Drilling (mo_9_)	*M*_1_ *,M*_2_ *,M*_3_	*CT*_1_	*TAD_−z_*
f10	A slot	Milling (mo_10_)	*M*_2_ *,M*_3_	*CT*_5_ *,CT*_6_	*TAD_−y_*
f11	A slot	Milling (mo_11_)	*M*_2_ *,M*_3_	*CT*_5_ *,_CT_*_7_	*TAD_−y_*
f12	Two holes as a replicated feature	Drilling (mo_12_)	*M*_1_ *,M*_2_ *,M*_3_	*CT*_1_	*TAD_+z_, TAD_−z_*
f13	A step	Milling (mo_13_)	*M*_2_ *,M*_3_	*CT*_5_ *,CT*_6_	*TAD_-x_, TAD_−y_*
f14	Two holes a replicated feature	Drilling (mo_14_)	*M*_1_ *,M*_2_ *,M*_3_	*CT*_1_	*TAD_−_* *_y_*

**Table 4:** Precedence relationships for prismatic part 2.

Manufacturing Interactions	Precedence Relationships	Type of Precedence Constraints
Tool-feature interaction	mo_1_ must be executed before mo_2_	Hard constraints
Feature-datum interactions	mo_6_ must be executed before mo_7_	
	mo_10_ must be executed before mo_11_	
	mo_13_ must be executed before mo_14_	
Thin-wall interactions	mo_9_ must be executed before mo_8_	Soft Constraints
	mo_12_ must be executed before mo_10_	
Material removal interactions	mo_8_ must be executed before mo_9_	
	mo_10_ must be executed before mo_12_	
	mo_13_ must be executed before mo_14_	
	mo_3_ must be executed before mo_4_	

**Table 5:** Information about features and resource candidates for prismatic part 3.

Feat.	Feature Definition	Machining Operations	Machines	Cutting Tools	TADs
f1	Pocket	Milling (mo_1_)	*M*_2_ *,M*_3_	*CT*_5_ *,CT*_6_ *,CT*_7_	*TAD_−z_*
f2	Blind hole	Drilling (mo_2_)	*M*_1_ *,M*_2_ *,M*_3_	*CT*_2_ *,CT*_3_ *,CT*_4_	*TAD_−z_*
f3	Through hole	Drilling (mo_3_)	*M*_1_ *,M*_2_ *,M*_3_	*CT*_2_ *,CT*_3_ *,CT*_4_	*TAD_+z_, TAD_−z_*
		Reaming (mo_4_)	*M*_1_ *,M*_2_ *,M*_3_	*CT*_9_	*TAD_+z_, TAD_−z_*
		Boring (mo_5_)	*M*_3_ *,M*_4_	*CT*_8_	*TAD_+z_, TAD_−z_*
f4	Four Through holes	Drilling (mo_6_)	*M*_1_ *,M*_2_ *,M*_3_	*CT*_2_	*TAD_+z_, TAD_−z_*
f5	Slot	Milling (mo_7_)	*M*_2_ *,M*_3_	*CT*_5_ *,CT*_6_	*TAD_-y_, TAD_−z_*
f6	Slot	Milling (mo_8_)	*M*_2_ *,M*_3_	*CT*_5_ *,CT*_6_	*TAD_+y_, TAD_−z_*
f7	Two Blind holes	Drilling (mo_9_)	*M*_1_ *,M*_2_ *,M*_3_	*CT*_1_	*TAD_+y_*
f8	Two slots	Milling (mo_10_)	*M*_2_ *,M*_3_	*CT*_6_ *,CT*_7_ *,CT*_10_	*TAD_+y_, TAD_−y_, TAD_+z_*
f9	Pocket	Milling (mo_11_)	*M*_2_ *,M*_3_	*CT*_5_ *,CT*_6_ *,CT*_7_	*TAD_−x_*
f10	Pocket	Milling (mo_12_)	*M*_2_ *,M*_3_	*CT*_5_ *,CT*_6_ *,CT*_7_	*TAD_+x_*
f11	Plane	Rough milling (mo_13_)	*M*_2_ *,M*_3_	*CT*_5_ *,CT*_6_ *,CT*_7_	*TAD_+x_, TAD_−x_, TAD_+y_,* *TAD_−y_, TAD_+z_*
		Finish milling (mo_14_)	*M*_2_ *,M*_3_	*CT*_5_ *,CT*_6_ *,CT*_7_	

**Table 6:** Available machining resources for three prismatic parts.

	Prismatic Part 1	Prismatic Part 2	Prismatic Part 3
	Machines		
*M*_1_	Drilling press	Drilling press	Drilling press
*M*_2_	3-axis vertical milling machine	Milling machine	3-axis vertical milling machine
*M*_3_	CNC 3-axis vertical milling machine	3-axis vertical milling machine	CNC 3-axis vertical milling machine
*M*_4_	Boring machine	-	Boring machine
	Tools		
*CT*_1_	Drill	Drill 1	Drill 1
*CT*_2_	Drill	Drill 2	Drill 2
*CT*_3_	Drill	Reamer	Drill 3
*CT*_4_	Drill	Boring tool	Drill 4
*CT*_5_	Tapping tool	Milling cutter 1	Milling cutter 1
*CT*_6_	Mill	Milling cutter 2	Milling cutter 2
*CT*_7_	Mill	Slot cutter	Milling cutter 3
*CT*_8_	Mill	Chamfer tool	Boring tool
*CT*_9_	Reamer	-	Reamer
*CT*_10_	Boring tool	-	Slot cutter

**Table 7:** Cost information for the considered prismatic parts.

Resources	Prismatic part 1									
Machines	*M*_1_	*M*_2_	*M*_3_	*M*_4_						
Machine cost index (*MCI*)	10	40	100	60						
Cutting tools	*CT*_1_	*CT*_2_	*CT*_3_	*CT*_4_	*CT*_5_	*CT*_6_	*CT*_7_	*CT*_8_	*CT*_9_	*CT*_10_
Cutting tool cost index (*CTCI*)	7	5	3	8	7	10	15	30	15	20
Change indices	*MCC*	*SCC*	*CTCC*							
Cost	160	100	20							
Resources	Prismatic part 2									
Machines	*M*_1_	*M*_2_	*M*_3_							
Machine cost index (*MCI*)	10	35	60							
Cutting tools	*CT*_1_	*CT*_2_	*CT*_3_	*CT*_4_	*CT*_5_	*CT*_6_	*CT*_7_	*CT*_8_		
Cutting tool cost index (*CTCI*)	3	3	8	15	10	15	10	10		
Change indices	*MCC*	*SCC*	*CTCC*							
Cost	300	120	15							
Resources	Prismatic part 3									
Machines	*M*_1_	*M*_2_	*M*_3_	*M*_4_						
Machine cost index (*MCI*)	10	40	100	60						
Cutting tools	*CT*_1_	*CT*_2_	*CT*_3_	*CT*_4_	*CT*_5_	*CT*_6_	*CT*_7_	*CT*_8_	*CT*_9_	*CT*_10_
Cutting tool cost index (*CTCI*)	7	5	3	8	7	10	15	30	20	15
Change indices	*MCC*	*SCC*	*CTCC*							
Cost	160	120	20							

**Table 8:** Optimal process plans for prismatic part 1 and three optimization conditions.

Condition 1																				
Machining operation	1	5	3	2	18	4	17	11	6	12	13	7	8	9	19	20	14	10	15	16
Machine	2	2	2	2	2	2	2	2	2	2	2	2	2	2	2	4	4	4	1	1
Cutting tool	6	6	6	6	6	2	7	7	7	4	9	7	4	9	9	10	10	10	1	5
TAD	*+z*	*+x*	*+x*	*−z*	*−z*	*−z*	*−z*	*−z*	*−z*	*−z*	*−z*	*−a*	*−a*	*−a*	*+z*	*+z*	*−z*	*−a*	*−z*	*−z*
TMC = 800, TMCC = 320, NMC = 2, TCTC = 250, TCTCC = 200, NCTC = 10, TSC = 900, NSC = 9, TWMC = **2470**																				
Condition 2																				
Machining operation	1	18	11	2	6	12	13	17	5	3	7	8	9	19	10	20	14	4	15	16
Machine	2	2	2	2	2	2	2	2	2	2	2	2	2	2	4	4	4	1	1	1
Cutting tool	8	7	8	8	8	3	9	8	7	8	8	3	9	9	10	10	10	2	1	5
TAD	*+z*	*−z*	*−z*	*−z*	*−z*	*−z*	*−z*	*−z*	*−z*	*+x*	*−a*	*−a*	*−a*	*+z*	*−a*	*+z*	*−z*	*−z*	*−z*	*−z*
TMC = 770, TMCC = 320, NMC = 2, TSC = 900, NSC = 9, TWMC = **1990**																				
Condition 3																				
Machining operation	1	3	18	17	5	11	2	6	12	13	14	19	20	7	8	9	10	4	15	16
Machine	3	3	3	3	3	3	3	3	3	3	3	3	3	3	3	3	3	1	1	1
Cutting tool	7	7	7	7	7	7	6	7	4	9	10	9	10	7	4	9	10	2	1	5
TAD	*+z*	*+x*	*−z*	*−z*	*−z*	*−z*	*−z*	*−z*	*−z*	*−z*	*−z*	*+z*	*+z*	*−a*	*−a*	*−a*	*−a*	*−z*	*−z*	*−z*
TMC = 1730, TMCC = 160, NMC = 1, TSC = 600, NSC = 6, TWMC = **2490**																				

**Table 9:** Comparative results of HECSA with other algorithms for three prismatic parts.

Algorithm	Condition 1			Condition 2			Condition 3		
	Mean	Max	Min	Mean	Max	Min	Mean	Max	Min
Prismatic part 1									
**HGWO**	**2608.9**	**2805**	**2470**	**2092**	**2240**	**1990**	**2499.5**	**2500**	**2490**
HGASA (Li et al. [5])	2546	2585	2527	2120	2120	2120	-	2600	2590
PSO (Guo et al. [42])	2680.5	-	2535	-	-	-	-	-	-
HybGA (Huang et al. [8])	-	-	2527	-	-	2120	-	-	2590
HBMO (Wen et al. [24])	2543.5	2557	2525	2098	2120	2090	2592.4	2600	2590
ACO (Wang et al. [15])	2456.1	2527	2435	2115.4	2380	2090	2600	2740	2580
TSACO (Wang et al. [16])	2552.4	2557	2525	2120.5	2380	2090	2600.8	2740	2590
cPSO (Petrović et al. [20])	2629	2687	2520	2100	2220	2020	2515	2600	2500
mACO (Hu et al. [17])	2666	-	2530	2115	-	2090	-	-	-
ESGA (Su et al. [11])	2539.1	2562	2530	-	-	2090	-	-	2590
IWD (Gao et al. [32])	2553.5	2554	2527	2123	2380	2090	2615.3	2740	2590
Prismatic part 2									
**HGWO**	**1344.3**	**1363**	**1328**	**1193**	**1290**	**1170**			
HybGA (Huang et al. [8])	1370	1583	1328	1224	1410	1170			
TS (Liu et al. [14])	1342	1378	1328	1194	1298	1170			
SA (Liu et al. [14])	1373.5	1518	1328	1217	1345	1170			
GA (Liu et al. [14])	1611	1778	1478	1482	1650	1410			
ACO (Liu et al. [14])	1329.5	1343	1328	1170	1170	1170			
cPSO (Petrović et al. [20])	1654	1748	1493	1428	1530	1270			
Prismatic part 3									
**HGWO**	**1918.8**	**2003**	**1815**						
HGASA (Li et al. [5])	1927.1	2079	1823						
PSO (Guo et al. [42])	2408.5	2280	2593						
GA (Kafashi [6])	-	-	2057						
HybGA (Huang et al. [8])	1828	1895	1749						
cPSO (Petrović et al. [20])	1871.5	1934	1775						

**Table 10:** Optimal process plans for prismatic part 2 for two conditions.

Condition 1														
Machining operation	8	5	3	4	13	10	11	14	12	6	1	7	9	2
Machine	2	2	2	2	2	2	2	2	2	2	2	2	2	2
Cutting tool	5	5	5	5	5	5	5	1	1	2	1	1	1	8
TAD	*+x*	*+y*	*+y*	*+y*	*−y*	*−y*	*−y*	*−y*	*−z*	*−z*	*−z*	*−z*	*−z*	*−z*
TMC = 490, TMCC = 0, NMC = 0, TCTC = 98, TCTCC = 60, NCTC = 4, TSC = 480, NSC = 4, TWMC = **1328**														
Condition 2														
Machining operation	8	1	6	12	7	9	5	3	4	13	14	2	10	11
Machine	2	2	2	2	2	2	2	2	2	2	2	2	2	2
Cutting tool	5	1	2	1	1	1	6	6	6	6	1	8	6	5
TAD	*+x*	*−z*	*−z*	*−z*	*−z*	*−z*	*+y*	*+y*	*+y*	*−y*	*−y*	*−y*	*−y*	*−y*
TMC = 490, TMCC = 0, NMC = 0, TSC = 480, NSC = 4, TWMC = **1170**														

**Table 11:** Optimal process plans for prismatic part 3 for two conditions.

Machining operation	11	13	12	14	8	9	10	7	1	3	5	4	2	6
Machine	2	2	2	2	2	2	2	2	2	2	3	3	3	3
Cutting tool	5	5	5	5	5	1	6	6	6	3	8	9	2	2
TAD	*−x*	*+x*	*+x*	*+x*	*+y*	*+y*	*+y*	*−z*	*−z*	*−z*	*−z*	*−z*	*−z*	*−z*
TMC = 800, TMCC = 160, NMC = 1, TCTC = 135, TCTCC = 120, NCTC = 6, TSC = 600, NSC = 5, TWMC = **1815**														

**Table 12:** Mean computational times (s) of five different algorithms for three experimental studies.

Algorithm	Prismatic Part 1			Prismatic Part 2		Prismatic Part 3
	Cond. 1	Cond. 2	Cond. 3	Cond. 1	Cond. 2	Cond. 1
**HGWO**	**67.9351**	**67.3275**	**89.2144**	**84.5813**	**86.2835**	**62.8624**
HybGA	42.1551	42.9072	47.7353	50.0639	50.6163	38.1999
PSO	70.3245	70.0847	90.9951	83.9863	84.4812	64.7519
GA	38.9688	38.2773	44.7899	43.3343	44.1306	34.0308
GWO	43.6161	42.2103	60.7819	48.9564	49.5188	38.3231
cPSO	75.7522	78.1318	97.5841	93.0888	93.8023	72.2037

**Table 13:** MATLAB profile summary report about the execution times of certain MATLAB functions.

Function Name (MATLAB)	Execution Times for the Several Functions Regarding Prismatic Part					
	Condition 1		Condition 2		Condition 3	
	Total Time	Self-Time	Total Time	Self-Time	Total Time	Self-Time
fitness_cost	3229.754	3229.754	3645.48	3645.48	3527.900	3527.900
cpso_algorithm	2005.366	722.229	2185.712	791.54	2061.431	771.305
**hgwo_algorithm**	**1823.554**	**583.585**	**2011.849**	**651.219**	**1895.894**	**638.906**
pso_algorithm	1791.113	701.895	2006.323	798.087	1928.071	766.405
constr_handling	1283.064	1283.064	1387.015	1387.015	1353.541	1353.541
gwo_algorithm	1231.354	535.619	1332.102	579.912	1303.338	569.971
hybga_algorithm	971.847	42.707	1085.238	47.323	1015.884	44.017
ga_algorithm	889.644	27.057	989.088	30.224	964.780	29.588

## Data Availability

Data sharing is not applicable to this article.
